# The Oxidation Cascade of a Rare Multifunctional P450 Enzyme Involved in Asperterpenoid A Biosynthesis

**DOI:** 10.3389/fchem.2021.785431

**Published:** 2021-12-16

**Authors:** Hui-Yun Huang, Jia-Hua Huang, Yong-Heng Wang, Dan Hu, Yong-Jun Lu, Zhi-Gang She, Guo-Dong Chen, Xin-Sheng Yao, Hao Gao

**Affiliations:** ^1^ Institute of Traditional Chinese Medicine and Natural Products, College of Pharmacy/Guangdong Province Key Laboratory of Pharmacodynamic Constituents of TCM and New Drugs Research, Jinan University, Guangzhou, China; ^2^ School of Traditional Chinese Materia Medica, Shenyang Pharmaceutical University, Shenyang, China; ^3^ School of Life Sciences, Sun Yat-sen University, Guangzhou, China; ^4^ School of Chemistry, Sun Yat-sen University, Guangzhou, China

**Keywords:** multifunctional P450s, methyl oxidation, asperterpenoids, *m*PTPB inhibition, oxidation cascade

## Abstract

The cytochrome P450 enzymes (P450s or CYPs) are heme-containing enzymes which catalyze a wide range of oxidation reactions in nature. In our previous study, a rare multifunctional P450 AstB was found, which can dually oxidize two methyl groups (C-19 and C-21) of preasperterpenoid A to asperterpenoid A with 3-carboxyl and 11-hydroxymethyl groups. However, the oxidation order of C-19 and C-21 catalyzed by AstB is unclear. In order to reveal this oxidation order, probable pathways catalyzed by AstB were proposed, and the oxidation order of C-19 and C-21 was obtained by quantum chemistry calculations. The potential intermediates (three new asperterpenoids D–F, **1**–**3**) were obtained through the chemical investigation on the extract of the transformant strain and chemical conversions, which were used as the standards to detect their existences in the extract of the transformant strain with HPLC-MS. Combined with the quantum chemistry calculation and the HPLC-MS analysis, the catalyzed order of AstB in asperterpenoid A biosynthesis was revealed. Furthermore, the *m*PTPB inhibition of obtained asperterpenoids was evaluated, and the results showed that 3-carboxyl and the oxidation station of C-21 would be the key factors for *m*PTPB inhibition of asperterpenoids.

## Introduction

Cytochrome P450 enzymes (P450s or CYPs) are a kind of enzyme catalyzing a wide range of oxidation reactions at the specific site of molecules, which play an important role in the metabolism of organisms (Sheng et al., 2009) and the biosynthesis of natural products with potent bioactivity (Jiang et al., 2021; Lin et al., 2019). For examples, human P450c11 catalyzes the generation of cortisol (one of glucocorticoids in human organisms) from 11-deoxycortisol (Bertram et al., 2012). P450 2D6 is in charge for the transformation of codeine to morphine (one of the famous analgesics) (Kramlinger et al., 2015). TwCYP712K1 performs the three-step oxidation of fridelin to polpunonic acid in celastrol (a potent anticancer and anti-obesity natural product from *Tripterygium wilfordii* Hook. f) (Zhou et al., 2021). More and more research studies have found that some P450s can be involved in the oxidation reactions with multiple sites of molecules (Bai et al., 2020; Child et al., 2019; Erickson et al., 2007; Yanni et al., 2008; Zeng et al., 2019) and catalyze non-oxidation reactions (Bai et al., 2020; Keyler et al., 2003; Long and Dolan, 2001; Peyronneau et al., 2012), which are classified as multifunctional P450s. In reported multifunctional P450 family, only few oxidation cascades have been demonstrated (Cochrane and Vederas, 2014; Erickson et al., 2007; Mendez et al., 2014; Moses et al., 2015; Narita et al., 2016; Zeng et al., 2019).

**FIGURE 1:**
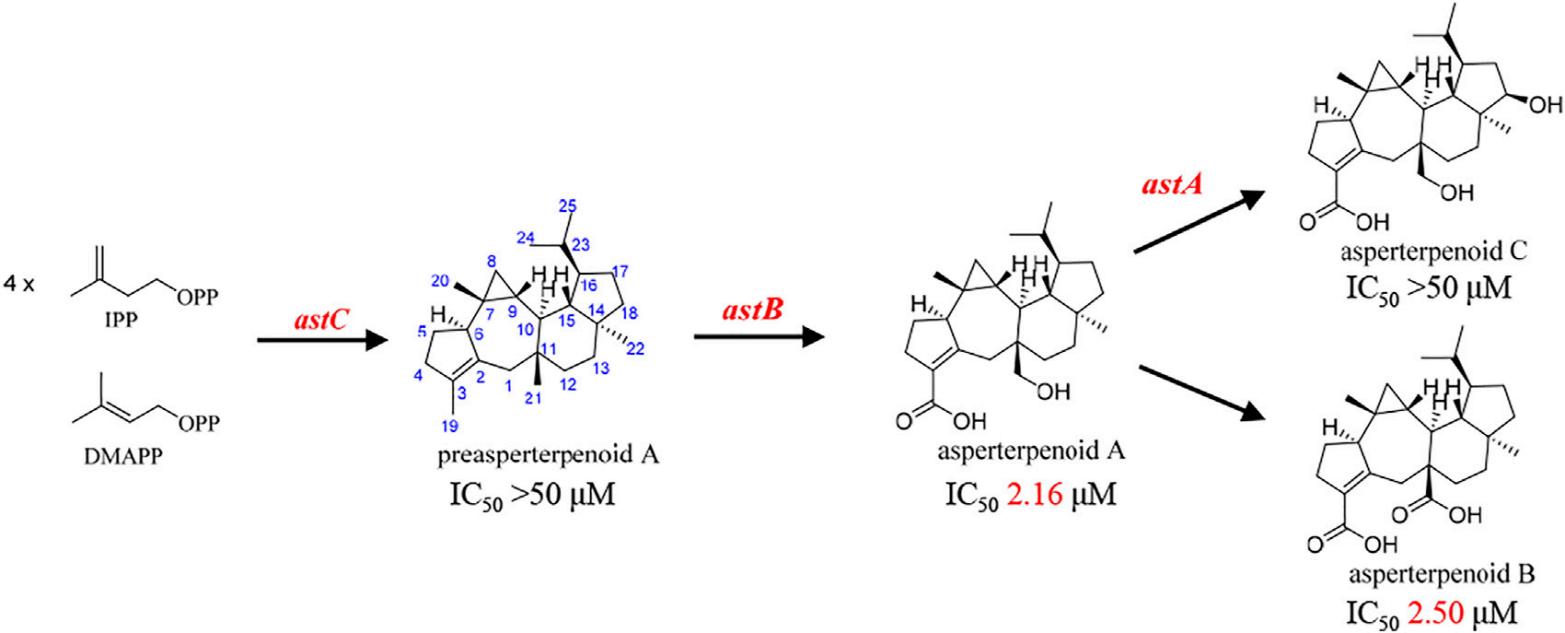
Biosynthesis and IC_50_ value (*m*PTPB inhibitions) of the reported asperterpenoids.

In our search of bioactive compounds from fungi through genome mining (Huang et al., 2019; Zhang et al., 2020), a rare oxidation multifunctional P450 AstB was found, which was solely responsible for the transformation of preasperterpenoid A to asperterpenoid A (the molecule with 3-carboxyl and 11-hydroxymethyl groups) *via* C-19 and C-21 oxidations of preasperterpenoid A (Figure 1). Furthermore, asperterpenoids A (IC_50_ = 2.16 μM) and B (the molecule with 3,11-dicarboxyl, IC_50_ = 2.50 μM) presented the potent inhibition against *Mycobacterium tuberculosis* protein tyrosine phosphatase B (*m*PTPB, a virulence factor secreted by *M. tuberculosis* and can facilitate the establishment of tuberculosis infection and pathogenesis) (Huang et al., 2013; Huang et al., 2019). However, the oxidation order of C-19 and C-21 catalyzed by AstB remained obscure because no intermediate in the generation of asperterpenoid A was obtained from the heterologously expressed *astBC*-harboring transformant strain (*Aspergillus oryzae*) with the previous fermentation condition (Huang et al., 2019). Therefore, the quantum chemistry calculations of the oxidation order of C-19 and C-21, the acquirements of the potential intermediates, and the HPLC-MS detection of the potential intermediates in the transformant strain were carried out. In addition, the *m*PTPB inhibition of obtained asperterpenoids was also evaluated.

**GRAPHICAL ABSTRACT:**
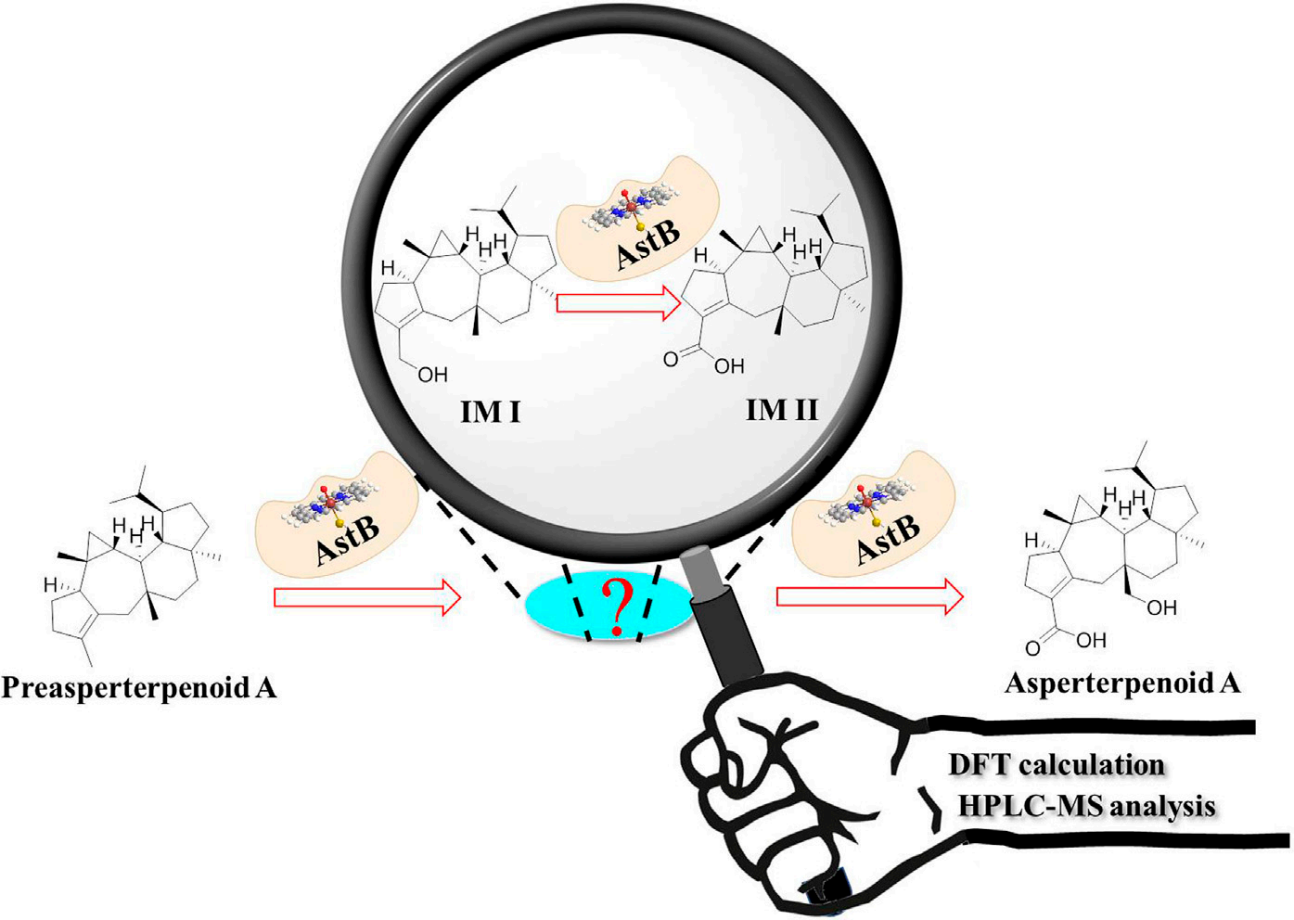


## Materials and Methods

### General Experimental Procedures

Methanol (MeOH) was purchased from Yuwang Industrial Co. Ltd. (Yucheng, China). Acetonitrile (MeCN) and acetone were obtained from Oceanpak Alexative Chemical Co. Ltd. (Gothenburg, Sweden). Cyclohexane and ethyl acetate (EtOAc) were analytical grade from Fine Chemical Co. Ltd. (Tianjin, China). The biochemical reagents and kits used in this study were purchased from TaKaRa Bio Inc. (Dalian, China), Thermo Fisher Scientific Inc. (Shenzhen, China), and Sangon Biotech Co. Ltd. (Shanghai, China), unless noted otherwise.

UV data, IR data, and optical rotations were, respectively, measured on the JASCO V-550 UV/vis spectrometer, JASCO FT/IR-4600 plus spectrometer, and JASCO P2000 digital polarimeter from JASCO International Co. Ltd. (Tokyo, Japan). ECD spectra were recorded in MeOH using a JASCO J-810 spectrophotometer (Jasco International Co. Ltd., Tokyo, Japan) at room temperature. The HRESIMS data were obtained on a Waters Micromass Q-TOF mass spectrometer from Waters Corporation (Milford, United States). 1D and 2D NMR spectra were recorded with the Bruker AV 600 spectrometer from Bruker BioSpin Group (Faellanden, Switzerland) using the solvent signals (CDCl_3_: *δ*_H_ 7.26/*δ*_C_ 77.0) as the reference. Analytical HPLC was performed on a Thermo Fisher HPLC system equipped with an Ultimate 3000 pump, an Ultimate 3000 diode array detector, an Ultimate 3000 column compartment, an Ultimate 3000 autosampler (Thermo Fisher, United States), and an Alltech (Grace) 2000ES evaporative light scattering detector (Alltech, United States) using a COSMOSIL 3C18-EB column (4.6 mm i.d. × 150 mm, 3 μm) with a linear gradient of 50–100% H_2_O (0.1% formic acid)-MeCN (0.1% formic acid) in 25 min followed by 100% MeCN (0.1% formic acid) for 35 min at 1 ml min^−1^. The semi-preparative HPLC was performed on an Ultimate 3000 HPLC system (Thermo Fisher) with a YMC-Pack ODS-A column (10.0 mm i.d. × 250 mm, 5 μm). Column chromatography (CC) was carried out on silica gels (200–300 mesh) (Qingdao Haiyang Chemical Group Corporation, Qingdao, China).

### Density Functional Theory Calculation of Hydrogen Abstraction Catalyzed by AstB

The computational reaction model (105 atoms) consisted of the two parts: 1) preasperterpenoid A and 2) Cpd Ι [see Figure 3, a brief P450 enzyme including a truncated heme and a thiolate axial ligand (SH-)]. Geometries for all the stationary points, including the reactant complex (RC), product complex (PC), and transition state (TS), were fully optimized in the gas phase using the M06 method in conjugation of the SDD(Fe)/6-31G*(C, H, O, N, and S) basis set (Fukui, 1981; Hratchian and Schlegel, 2004). (For details, see Supplementary Information S1.)

### Fungal Source, Fermentation Condition Investigation, Extraction, and Isolation of Asperterpenoid D (1)

The strain of the *A. oryzae* transformant harboring *astBC* was obtained in previous study (Huang et al., 2019). The fungal strain was inoculated into 10 ml DPY medium (2% dextrin, 1% polypeptone, 0.5% yeast extract, 0.05% MgSO_4_·7H_2_O, 0.5% KH_2_PO_4_, and 0.01% adenine) and was cultured at 28°C and 200 rpm for 2 days as the seed broth. Then the broth was transferred into 15 Erlenmeyer flasks (500 ml), each containing 100 ml of fermentation medium and grown at 28°C and 200 rpm. The screening of the fermentation conditions was carried out through bifactor analysis with culture media (rice, ME, PDB, GPY, and maltose media) and fermentation days (3, 5, and 7 days for liquid media and 10, 20, and 45 days for the rice medium, respectively) as variable factors. The rice medium contained 70 g of rice and 105 ml distilled H_2_O on each flask; ME medium contained 2% malt extract, 1% polypeptone, and 2% starch; PDB medium contained 20% potato and 2% dextrose; GPY medium contained 2% starch, 0.5% peptone, and 0.2% yeast extract; maltose medium contained 3% starch, 0.15% yeast extract, 0.1% MgSO_4_, 0.25% malt extract, 0.2% KH_2_PO_4_, and 0.4% CaCO_3_ (Supplementary Figures S6–S10). Through the screening of fermented conditions, the intermediate II (asperterpenoid D, **1**) was found in rice, GPY, PDB, ME, and maltose media, respectively (Supplementary Figures S6–S11, Figure 4). Then fermentation was carried out in 30 Erlenmeyer flasks (500 ml), each containing 100 ml of ME medium. After autoclaving at 121°C for 30 min, each flask was inoculated with 10 ml of the seed broth and cultured at 28°C and 200 rpm for 3 days.

Mycelia were harvested by filtration and extracted with acetone (2 L). The extraction was repeated for three times, and the extract was dried under reduced pressure to obtain a crude extract (4.7 g). Then the crude extract was fractionated in a dried column vacuum chromatography system filled with silica gel, using cyclohexane (100%), cyclohexane-AcOEt (98:2), cyclohexane-AcOEt (90:10), AcOEt (100%), and MeOH (100%) to obtain fractions of 126.8, 20.5, 676.3, 544.1, and 700.6 mg, respectively. The fraction eluted with cyclohexane-AcOEt (98:2) was subjected to semi-preparative HPLC, using MeCN-H_2_O (90:10, v/v) containing 0.1% formic acid at a flow rate of 3 ml min^−1^ to yield asperterpenoid D (**1**) (*t*_R_: 13.8 min, 7.9 mg).

### The Preparations of Asperterpenoids E (2) and F (3)

Asperterpenoid E (IM-I, **2**) preparation: a magnetically stirred mixture of asperterpenoid D (**1**) (20 mg, 0.05 mmol) in dry THF (tetrahydrofuran) (10 ml) was treated with LiAlH_4_ (18.89 mg, 0.50 mmol), and the ensuing gray suspension was stirred at 85°C under a balloon of nitrogen for 48 h then extracted with water/ethyl acetate. The organic layer was concentrated under reduced pressure, and the residue was isolated by semi-preparative HPLC (YMC-Pack ODS-A column, 3 ml min^−1^) with isocratic elution of 85% MeCN-H_2_O containing 0.1% formic acid to yield **2** (*t*_R_: 62.0 min, 5.0 mg).

Asperterpenoid F (IM-IV, **3**) preparation: A magnetically stirred mixture of asperterpenoid A (40 mg, 0.10 mmol) in dry THF (10 ml) was treated with LiAlH_4_ (36.22 mg, 0.96 mmol), and the ensuing gray suspension was stirred at 85°C under a balloon of nitrogen for 48 h then extracted with water/ethyl acetate. The organic layer was concentrated under reduced pressure, and the residue was isolated by semi-preparative HPLC (YMC-Pack ODS-A column, 3 ml min^−1^) with isocratic elution of 85% MeCN-H_2_O containing 0.1% formic acid to yield **3** (*t*_R_: 27.0 min, 17.7 mg).

### Structural Characterizations of 1–3

Asperterpenoid D (**1**): amorphous white powder; ^1^H and ^13^C NMR (see Table 1); [α]^24^_D_ +85.5 (*c* 0.20, MeOH); UV (MeOH) λ_max_ (log *ε*) 203 (3.79) and 235 (3.96); ECD λ_nm_ (Δε) (MeOH) 211 (+1.46) and 243 (−2.84) nm; IR (KBr) *ν*_max_ 3,278, 3,049, 2,956, 2,927, 2,870, 2,837, 1,740, 1,679, 1,629, 1,458, 1,383, 1,287, 1,267, 948, and 936 cm^−1^; HRESIMS (positive) *m/z* 371.2934 [M + H]^+^ (calcd. for C_25_H_39_O_2_, 371.2950) (Supplementary Table S2, Supplementary Figures S14–S23).

**TABLE 1:** ^1^H (600 MHz) and ^13^C NMR (150 MHz) data of compounds **1**–**3** in CDCl_3_.

	1		2		3	
Position	*δ*_C_, type	*δ*_H_, (*J* in Hz)*^a^*	*δ*_C_, type	*δ*_H_, (*J* in Hz)*^a^*	*δ*_C_, type	*δ*_H_, (*J* in Hz)*^a^*
1	47.9, CH_2_	a: 3.57, d (13.4)	47.0, CH_2_	a: 2.44, d (13.9)	41.6, CH_2_	a: 2.94, d (13.7)
	—	b: 1.79, d (13.4)	—	b: 1.71, d (13.9)	—	b: 1.45, d (13.7)
2	162.1, C	—	134.7, C	—	135.6, C	—
3	126.4, C	—	140.6, C	—	140.7, C	—
4	33.1, CH_2_	a: 2.63	33.7, CH_2_	a: 2.48	35.5, CH_2_	a: 2.63
	—	b: 2.55, br dd (16.0, 9.8)	—	b: 2.33	—	b: 2.13, ddd (15.8, 9.6, 2.6)
5	26.0, CH_2_	a: 1.98, br dd (13.0, 7.5)	26.2, CH_2_	a: 1.97, br dd (13.1, 7.4)	26.6, CH_2_	a: 1.98. br dd (13.1, 7.2)
	—	b: 1.90, dq (13.0, 9.4)	—	b: 1.85, dq (13.0, 9.4)	—	b: 1.86, dq (13.1, 9.4)
6	56.8, CH	2.30, br d (8.7)	54.6, CH	2.17, br d (9.0)	55.4, CH	2.18, br d (8.8)
7	21.5, C	—	22.1, C	—	22.6, C	—
8	25.5, CH_2_	a: 0.61, dd (8.4, 4.2)	25.1, CH_2_	a: 0.55, dd (8.3, 4.2)	25.8, CH_2_	a: 0.60, dd (8.4, 4.3)
	—	b: 0.37, br t (4.7)	—	b: 0.33, dd (5.2, 4.2)	—	b: 0.33, br t (4.9)
9	29.8, CH	0.22	29.4, CH	0.10	29.1, CH	0.06, ddd (10.5, 8.4, 5.6)
10	47.7, CH	1.21	47.2, CH	1.19	47.7, CH	1.34, t (11.1)
11	40.4, C	—	39.2, C	—	43.9, C	—
12	39.3, CH_2_	a: 1.61	39.3, CH_2_	a: 1.60	30.0, CH_2_	a: 1.91
	—	b: 1.39	—	b: 1.33	—	b: 1.24
13	35.9, CH_2_	a: 1.40	35.9, CH_2_	a: 1.45	35.7, CH_2_	a: 1.47
	—	b: 1.31	—	b: 1.31	—	b: 1.27
14	43.0, C	—	42.9, C	—	42.8, C	—
15	50.9, CH	1.21	51.0, CH	1.19	51.4, CH	1.16, t (11.0)
16	45.4, CH	1.77	45.3, CH	1.76	45.6, CH	1.73, tdd (10.5, 4.3, 3.1)
17	22.4, CH_2_	a: 1.60	22.2, CH_2_	a: 1.61	22.2, CH_2_	a: 1.60
	—	b: 1.45	—	b: 1.45	—	b: 1.44
18	40.1, CH_2_	a: 1.37	40.0, CH_2_	a: 1.35	39.9, CH_2_	a: 1.37
	—	b: 1.00	—	b: 0.99	—	b: 1.00
19	171.6, C	—	59.0, CH_2_	a: 4.25, d (11.1)	59.2, CH_2_	a: 4.40, d (12.5)
	—	—	—	b: 4.17, d (11.1)	—	b: 3.98, d (12.5)
20	20.8, CH_3_	0.93, s	20.7, CH_3_	0.85, s	21.0, CH_3_	0.95, s
21	20.1, CH_3_	0.94, s	20.4, CH_3_	0.90, s	61.3, CH_2_	a: 3.69, d (10.9)
	—	—	—	—	—	b: 3.62, d (10.9)
22	17.8, CH_3_	0.74, s	17.6, CH_3_	0.73, s	17.7, CH_3_	0.76, s
23	28.5, CH	2.30	28.4, CH	2.33	28.3, CH	2.27
24	23.3, CH_3_	0.86, d (6.6)	23.2, CH_3_	0.86, d (6.6)	23.1, CH_3_	0.85, d (6.9)
25	15.3, CH_3_	0.78, d (6.6)	15.1, CH_3_	0.76, d (6.6)	15.0, CH_3_	0.74, d (6.9)

a Indiscernible signals from overlap or the complex multiplicity are reported without designating multiplicity.

Asperterpenoid E (**2**): amorphous white powder; ^1^H and ^13^C NMR (see Table 1); [*α*]^24^_D_ +64.0 (*c* 0.20, MeOH); UV (MeOH) λ_max_ (log *ε*) 203 (4.03) and 252 (4.27); ECD λ_nm_ (Δε) (MeOH) 208 (+2.19), 236 (−0.35), 263 (+4.08), and 330 (−1.60) nm; IR (KBr) *ν*_max_ 3,411, 2,955, 2,927, 2,871, 2,855, 1,704, 1,602, 1,460, and 1,384 cm^−1^; HRESIMS (positive) *m/z* 339.3036 [M + H−H_2_O]^+^ (calcd. for C_25_H_39_, 339.3046). (Supplementary Table S4, Supplementary Figures S26–S35).

Asperterpenoid F (**3**): amorphous white powder; ^1^H and ^13^C NMR (see Table 1); [*α*]^24^_D_ +36.5 (*c* 0.23, MeOH); UV (MeOH) λ_max_ (log *ε*) 208 (3.17) and 257 (3.59); ECD λ_nm_ (Δε) (MeOH) 207 (+2.76), 233 (−0.03), 261 (+0.96), and 338 (−0.43) nm; IR (KBr) *ν*_max_ 3,316, 2,953, 2,930, 2,890, 1,672, 1,461, 1,379, and 1,023 cm^−1^; HRESIMS (positive) *m/z* 395.2917 [M + Na]^+^ (calcd. for C_25_H_40_O_2_Na, 395.2921) (Supplementary Table S5, Supplementary Figures S36–S45).

## Results and Discussion

**FIGURE 2:**
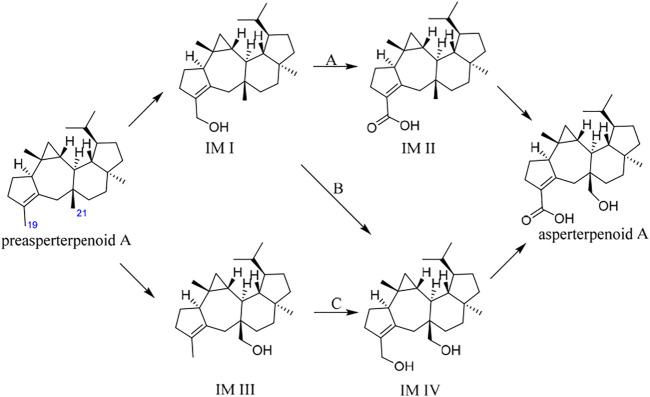
Three possible routes for AstB enzyme catalyzing oxidations.

It is known that hydroxymethyl is a common intermediate in oxidation of a methyl group to carboxyl, such as 12-OH-(-)-JAMe (the intermediate derived from (-)-JAMe) as the key intermediate in the generation of 12-COOH-(-)-JAMe (Kombrink, 2012). Because the oxidation orders of 3- and 11-methyls to hydroxymethyls and 3-hydroxymethyl to 3-carboxyl are unclear, there would be four potential intermediates, leading to three possible oxidation routes (A, B, and C) for the generation of asperterpenoid A from preasperterpenoid A catalyzed by AstB (Figure 2).

To the best of our knowledge, the hydrogen atoms of allyl carbons are generally more active and easier to leave than those of other kinds of carbons (Zerth et al., 2003). Based on this, it is proposed that the route A would exist in the transformation of preasperterpenoid A to asperterpenoid A catalyzed by AstB because C-19 (allyl carbon) oxidation is theoretically prior to C-21 oxidation in the route A. In order to confirm this inference, the DFT calculations were carried out (Denningtonll TK et al., 2003; Frisch GWT et al., 2013; Fukui, 1981; Hratchian and Schlegel, 2004; Sansen et al., 2007; Tao et al., 2015).

**FIGURE 3:**
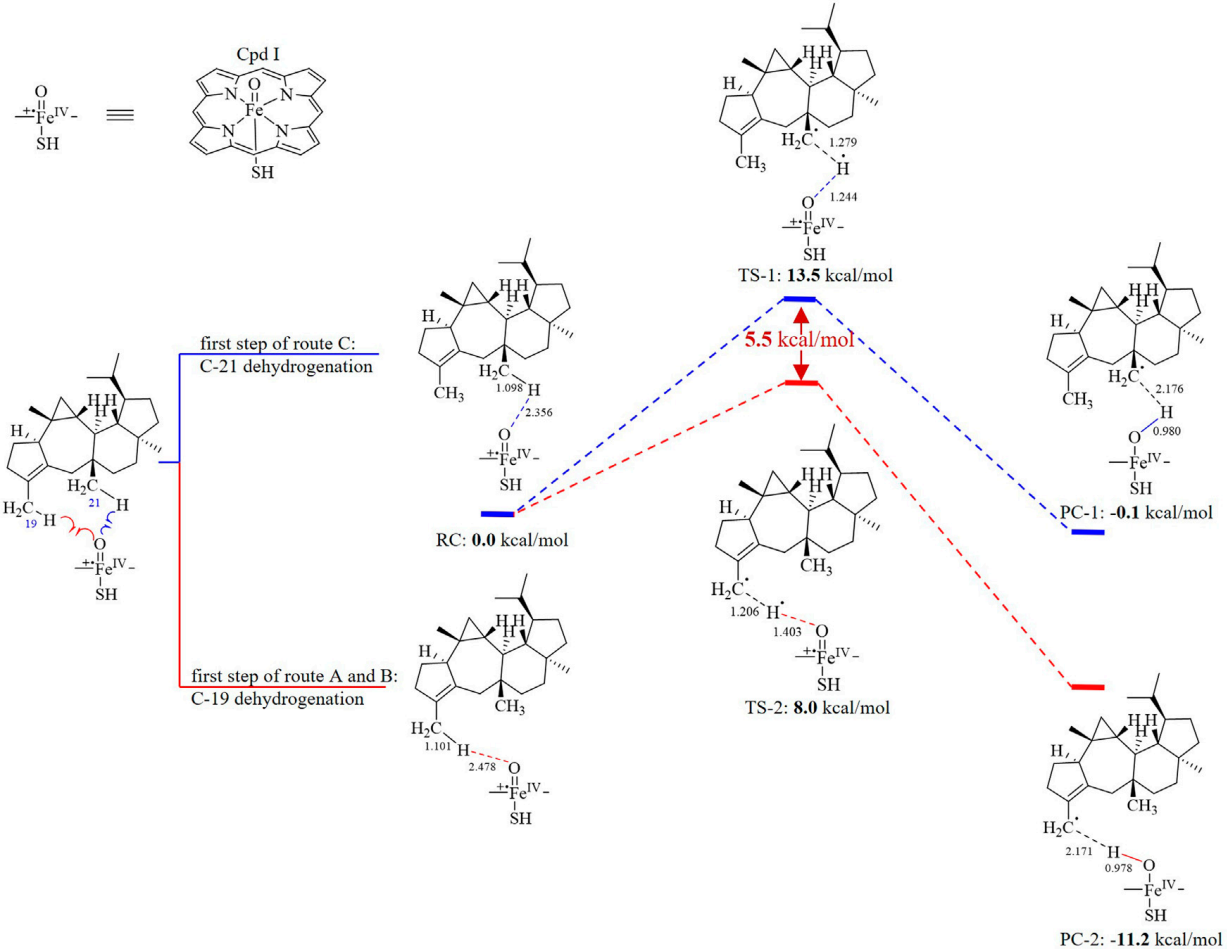
Dehydrogenation energy calculations of C-19 dehydrogenation (first step of route A and B) and C-21 dehydrogenation (first step of route C).

The oxidation first occurring at C-19 or C-21 is decided by the Gibbs free energy barriers of C-19 and C-21 dehydrogenations. Based on the DFT calculations (Figure 3), the free energy barriers of C-19 (transition state 2, TS-2) and C-21 (transition state 1, TS-1) dehydrogenations were predicted to be 8.0 and 13.5 kcal/mol, respectively. Therefore, the energy gap of two dehydrogenations was 5.5 kcal/mol (the probability of the occurrence of C-19 dehydrogenation was 99.99%), which indicated that C-19 oxidation would be the first step in the oxidations catalyzed by AstB. The calculation results also confirmed that the hydrogen atoms of allyl carbons are easier to leave than those of other kinds of carbons. In addition, H-19 in IM I would be easier to leave rather than H-21 because of the inductive effect from 19-hydroxyl group. Therefore, the 3-hydroxymethyl group of IM I would be subsequently oxidized to form IM II, rather than to form IM IV. These results were consistent with our inference that the route A would exist in the oxidations of AstB. (For details, see Supplementary Information S1).

**FIGURE 4:**
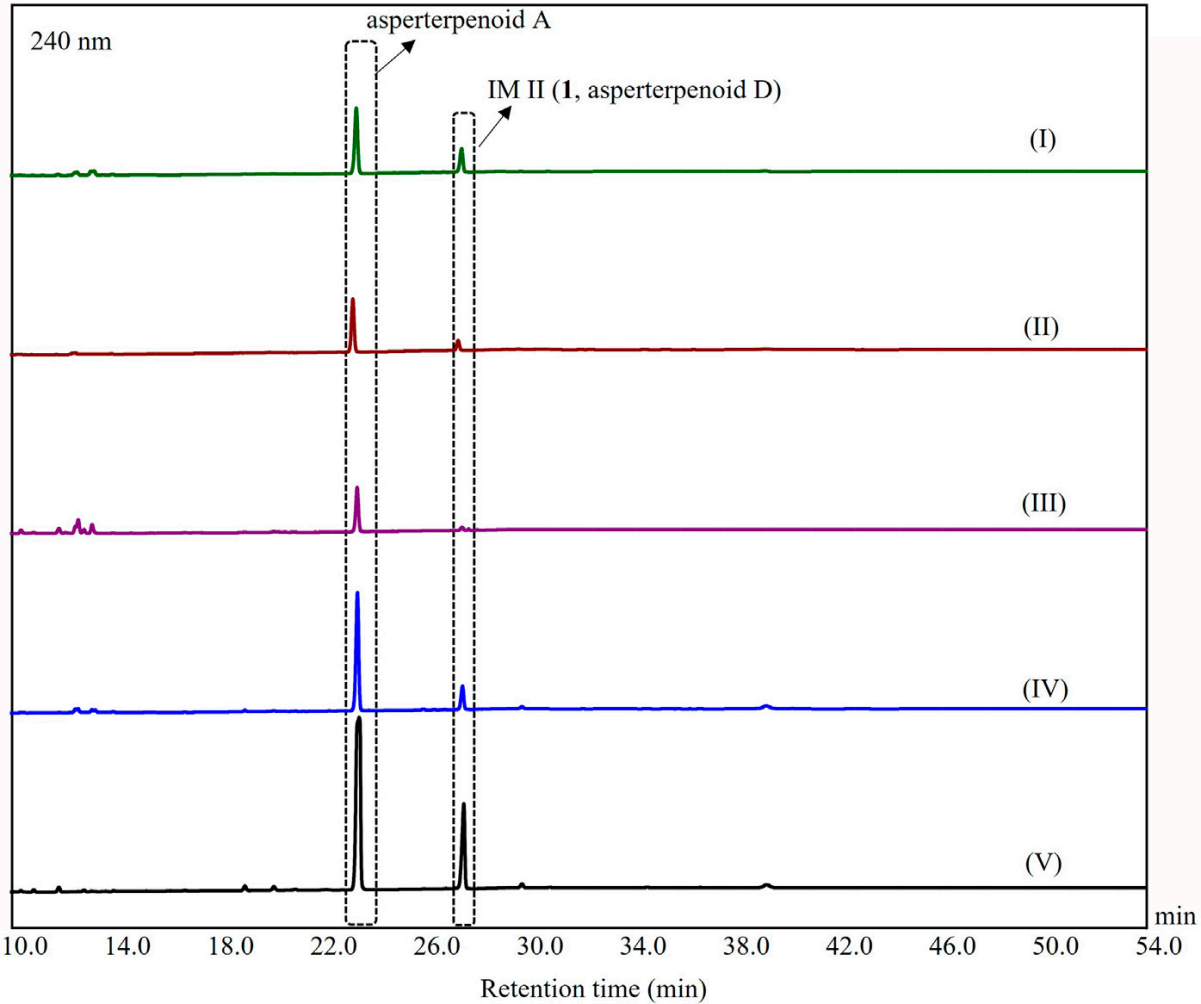
HPLC analysis of metabolites from *A. oryzae* transformants harboring *astBC*. (I) The strain was cultured in the GPY medium for 3 days, (II) the strain was cultured in the PDB medium for 3 days, (III) the strain was cultured in the rice medium for 10 days, (IV) the strain was cultured in the maltose medium for 3 days, and (V) the strain was cultured in the ME medium for 3 days.

In order to confirm the results from the DFT calculations, the acquirement of the key intermediates was necessary. In our previous study, no intermediate was observed in the extract of the heterologous expressed *astBC*-harboring transformant strain (*A. oryzae*), which would be resulted from the fermentation without an appropriate condition or the trace amount of the intermediates (Huang et al., 2019). According to this, the screening of the fermentation conditions was carried out using a bifactor analysis with a culture medium (rice, GPY, PDB, ME, and maltose media) and fermentation days (3, 5, and 7 days for liquid media and 10, 20, and 45 days for rice medium) as variable factors. (For details, see Supplementary Information S2, Supplementary Figures S6–S10.) An additional chromatographic peak was observed when the strain was fermented with rice, GPY, PDB, maltose, and ME media, respectively. Among them, the peak in the extract of the fermentation using ME medium with 3 days was the most obvious (Figure 4). The HPLC-MS analysis showed that the peak would be a new asperterpenoid (the positive ion peak at *m/z* 353.39 [M + H − H_2_O]^+^) (Supplementary Figure S11). After the isolation of this additional chromatographic peak through the large-scale fermentation with the ME medium for 3 days, the peak compound (**1**) was obtained. Based on the detailed NMR analysis (Supplementary Table S2, Supplementary Figures S18–S23) combined with ECD calculation (Supplementary Information S4.2, Supplementary Figures S24, S25), the structure of **1** was determined as a new asperterpenoid with 3-methyl oxidized to 3-carboxyl, which was IM II in route A and named as asperterpenoid D. The result confirmed the existence of route A in the oxidations catalyzed by AstB.

**FIGURE 5:**
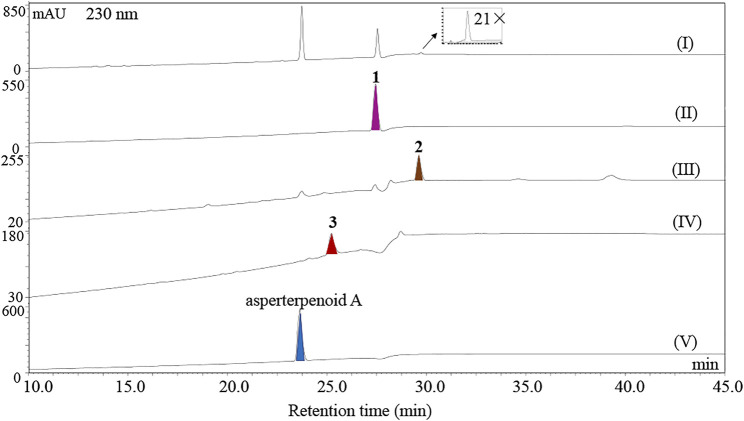
HPLC analysis of metabolites. (I) HPLC chromatogram of the extract of the *A. oryzae* transformants harboring *astBC* cultured in the ME medium for 3 days, (II–V) HPLC chromatograms of asperterpenoid D (**1**), asperterpenoid E (**2**), asperterpenoid F (**3**), and asperterpenoid A.

**FIGURE 6:**
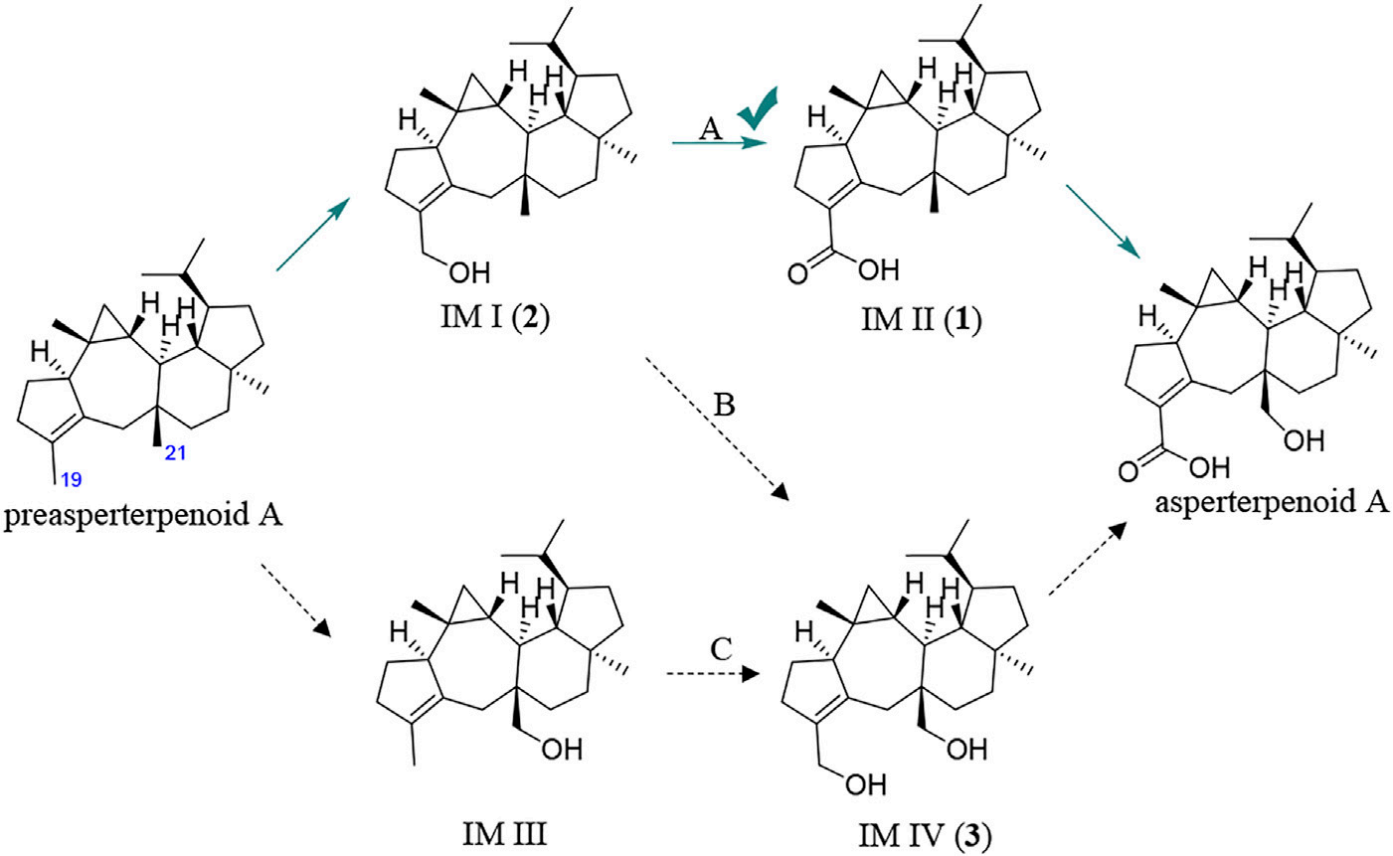
Main pathway for AstB enzyme catalyzing oxidations.

Furthermore, the potential intermediates IM I and IM IV were prepared with the artificial reduction from asperterpenoid D and asperterpenoid A because the potential intermediates IM I and IM IV were the biosynthetic precursors of asperterpenoid D and asperterpenoid A in the relevant proposed route. After reduction from asperterpenoid D with LiAlH_4_, the potential intermediate IM I (**2**, named asperterpenoid E) was obtained, and its structure was confirmed as a new asperterpenoid with 3-methyl oxidized to 3-methylol based the detailed analyses of NMR data (Supplementary Table S4, Supplementary Figures S26–S35). By the same way, the potential intermediate IM IV (**3**, named asperterpenoid F) was also obtained, and its structure was identified by the detailed analyses of NMR (Supplementary Table S5, Supplementary Figures S36–S45). Through HPLC-MS analysis with compounds **1**–**3** and asperterpenoid A as standards (HPLC analysis see Figure 5, HPLC-MS analysis see Supplementary Figures S12, S13), a tiny chromatographic peak (*t*_R_ = 29.6 min) was identified as asperterpenoid E (**2**) in the HPLC chromatogram of the *astBC*-harboring transformant strain (*A. oryzae*) in the ME medium cultured for 3 days (see Figure 5I, Supplementary Information S3, Supplementary Figure S12). In the HPLC-MS analysis, however, there was no peak with the same retention time and ion peak as asperterpenoid F (**3**) in the extract of the *astBC*-harboring transformant strain (*A. oryzae*) (see Supplementary Figure S13), indicating that **3** would not exist. These experiment data supported that the route A was the oxidization cascade of AstB (Figure 6).

**FIGURE 7:**
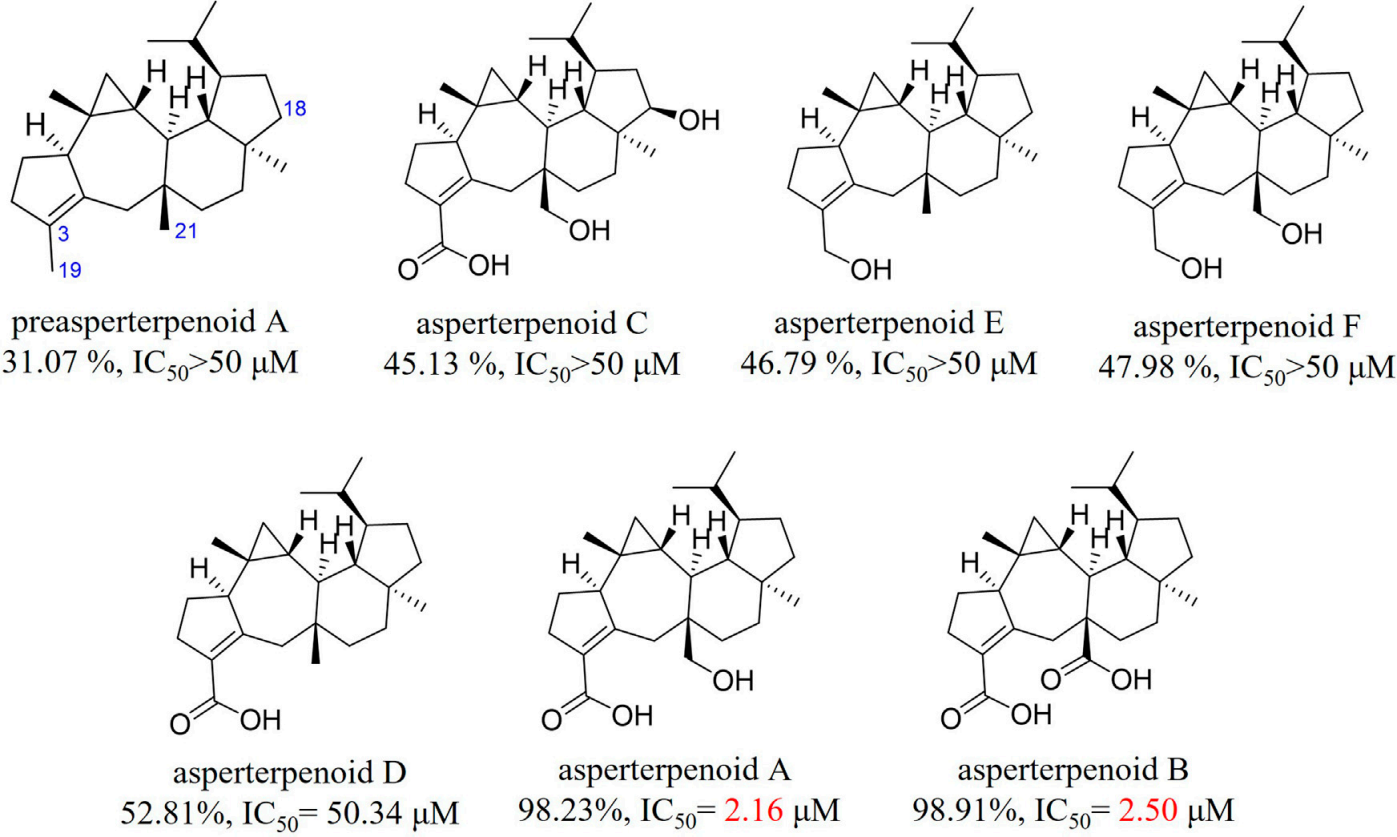
*m*PTPB inhibition at 50 μM and IC_50_ value of asperterpenoids.

In the previous study, it was found that asperterpenoids A and B showed potent inhibition against *m*PTPB and 18-hydroxyl would weaken the inhibition (Huang et al., 2019). For the abstention of asperterpenoids with 3-hydroxymethyl, the relationship between the oxidation stations of C-19 and C-21 in asperterpenoids and their *m*PTPB inhibition was unknown. In this research, the *m*PTPB inhibition of three new asperterpenoids (**1**–**3**, asperterpenoids D–F) and five known asperterpenoids (preasperterpenoid A and asperterpenoids A–C) was evaluated (Figure 7, detail see Supplementary Information S7, Supplementary Table S6). Among these new asperterpenoids, only asperterpenoid D (**2**) presented the *m*PTPB inhibition (IC_50_ = 50.34 μM), which was weaker than those of asperterpenoids A and B. The data clearly showed that 3-carboxyl and the oxidation station of C-21 were essential for *m*PTPB inhibition of asperterpenoids.

In conclusion, the oxidation cascade of a rare multifunctional P450 enzyme (AstB) was cleared based on the combination of the quantum chemistry calculations and the experiments of obtaining the potential intermediates and the HPLC-MS detection of the potential intermediates. Furthermore, the relationship between the oxidation stations of C-19 and C-21 in asperterpenoids and their *m*PTPB inhibition was also revealed.

## Data Availability

The original contributions presented in the study are included in the article/Supplementary Material; further inquiries can be directed to the corresponding authors.
