# Supplementary material for: The Oxidation Cascade of a Rare Multifunctional P450 Enzyme Involved in Asperterpenoid A Biosynthesis
Source: Front Chem. 2021 Dec 16;9:785431. doi: 10.3389/fchem.2021.785431 (PMC8717867; doi:10.3389/fchem.2021.785431)
Supplement: Supplementary file 1 [file DataSheet1.docx]

Supplementary Material

*Supporting Information*

The oxidation cascade of a rare multifunctional P450 enzyme involved in asperterpenoid A biosynthesis

Hui-Yun Huang^1^, Jia-Hua Huang^2^, Yong-Heng Wang^1^, Dan Hu^1^, Yong-Jun Lu^3^, Zhi-Gang She^4^, Guo-Dong Chen^1*^, Xin-Sheng Yao^1,2^, Hao Gao^1*^

^1^Institute of Traditional Chinese Medicine and Natural Products, College of Pharmacy/Guangdong Province Key Laboratory of Pharmacodynamic Constituents of TCM and New Drugs Research, Jinan University, Guangzhou 510632, People’s Republic of China.

^2^ School of Traditional Chinese Materia Medica, Shenyang Pharmaceutical University, Shenyang 110016, China.

^3^School of Life Sciences, Sun Yat-sen University, Guangzhou 510275, People’s republic of China.

^4^School of Chemistry, Sun Yat-sen University, Guangzhou 510006, People’s republic of China.

*** Correspondence:**Corresponding Author Tel.: +86-20-85221559. Fax: +86-20-85221559. E-mail: tghao@jnu.deu.cn; chgdtong@163.com

Contents

[1 Quantum chemical calculations of energy barriers at C-19 dehydrogenation and C-21 dehydrogenation 3](#_Toc86351278)

[2 Screening of fermented conditions for *A. oryzae* transformant strain 23](#_Toc86351279)

[3 HPLC-MS analysis of *A. oryzae* transformant strain 28](#_Toc86351280)

[4 Structural elucidation of asperterpenoid D (**1**) 30](#_Toc86351281)

[4.1 NMR assignments, UV, IR, ECD, and HRESIMS for asperterpenoid D (**1**) 30](#_Toc86351282)

[4.2 Conformational analysis and ECD-calculation of asperterpenoid D (**1**) 36](#_Toc86351293)

[5 Structural elucidation of asperterpenoid E (**2**) 38](#_Toc86351296)

[6 Structural elucidation of asperterpenoid F (**3**) 45](#_Toc86351307)

[7 Heterologous expression, purification, and inhibition assay for *m*PTPB 51](#_Toc86351318)

# Quantum chemical calculations of energy barriers at C-19 dehydrogenation and C-21 dehydrogenation

The computational reaction model (105 atoms) consisted of the two parts: (a) preasperterpenoid A and (b) Cpd I (see Figure 3, a brief P450 enzyme including a truncated heme and a thiolate axial ligand (SH-)). All DFT calculations were carried out with the Gaussian 09 suite of programs. Geometries for all the stationary points, including the reactant complex (RC), product complex (PC), and transition state (TS), were fully optimized in the gas phase using the method m06 in conjugation of the SDD(Fe)/6-31G*(C, H, O, N, S) basis set. Transition states were affirmed by harmonic frequency analysis to possess only one imaginary frequency and the stationary points were confirmed as minima with all points. The connectivity between the stationary points was established by intrinsic reaction coordinate (IRC) calculations.

Based on the proposed pathways, C-19 and C-21 dehydrogenations were recognized as the beginning of the route A/B and route C, respectively.

**C-19 dehydrogenation** The optimized geometry of preasperterpenoid A-heme complex RC was concerted in the gas phase. The Fe‒O distance in RC was 1.624 Å, while the lengths of O‒H-19 bond and C-19‒H-19 bond were 2.478 Å and 1.101 Å, respectively. The located transition state TS-2 was characterized by its single imaginary frequency of 1117.86i cm^-1^, mainly corresponding to the proton (H) transferring from C-19 carbon atom to the O atom of Cpd Ι. In TS-2, the Fe‒O distance in TS-2 was 1.725 Å, while the length of O‒H-19 bond was reasonably reduced at 1.403 Å and the length of C-19‒H-19 bond was reasonably raised at 1.206 Å. The hydrogen atom was getting closer to the oxygen atom. The free energy barrier of RC to TS-2 was calculated as 8.0 kcal/mol. In PC-2, the Fe‒O distance in TS-2 was 1.799 Å, while the length of O‒H-19 bond was continuously reasonably reduced at 0.978 Å and the length of C-19‒H-19 was reasonably raised at 2.171 Å. This suggested that the hydrogen atom was extracted from the 19-carbon atom and combined with Cpd Ι.

**C-21 dehydrogenation** The optimized geometry of preasperterpenoid A-heme complex RC was concerted in the gas phase. The Fe-O distance in RC was 1.624 Å, while the lengths of O‒H-21 bond and C-21‒H-21 bond were 2.356 Å and 1.098 Å, respectively. The located transition state TS-1 was characterized by its single imaginary frequency of 2123.4i cm^-1^, mainly corresponding to the proton (H) transfer from C-19 carbon atom to the O atom of Cpd Ι. In TS-1, the Fe‒O distance in TS-1 was 1.761 Å, while the length of O‒H-21 bond was reasonably reduced at 1.244 Å and the length of C-21‒H-21 bond lengthened was reasonably raised at 1.279 Å. The hydrogen atom was getting closer to the oxygen atom. The free energy barrier of RC to TS-1 was calculated as 13.5 kcal/mol. In PC-1, the Fe‒O distance in TS-1 was 1.811 Å, while the length of O‒H-21 bond was continuously reasonably reduced at 0.980 Å and the length of C-21‒H-21 was reasonably raised at 2.176 Å. This suggested that the hydrogen atom was extracted from the 21-carbon atom and combined with Cpd Ι.

Figure **S1**| Reactant complex (RC) module. Bond lengths in Fe‒O, O‒H-21, C-21‒H-21 (left) and Fe‒O, O‒H-19, C-19‒H-19 (right)

Figure **S2**| Transition state 1 (TS-1) module. Bond lengths in Fe‒O, O‒H-21, C-21‒H-21

Figure **S3**| Transition state 2 (TS-2) module. Bond lengths in Fe‒O, O‒H-19, C-19‒H-19

Figure **S4**| Product complex 1 (PC-1) module. Bond lengths in Fe‒O, O‒H-21, C-21‒H-21

Figure **S5**| Product complex 2 (PC-2) module. Bond lengths in Fe‒O, O‒H-19, C-19‒H-19

The atomic coordinates of RC module

C -6.47871 -1.70542 2.55273

C -5.34125 -0.91098 1.91688

C -5.77191 -0.35344 0.54631

C -6.96389 0.64319 0.65771

C -6.51816 1.92979 -0.0475

C -5.50484 1.44996 -1.08299

C -6.26669 0.82467 -2.25704

C -4.53918 2.51667 -1.57913

C -3.42585 1.88391 -2.4036

C -2.66217 0.71181 -1.73799

C -1.67504 1.27484 -0.71386

C -1.88073 0.06253 -2.90893

C -1.04282 -1.14652 -2.67741

C 0.28484 -1.28617 -2.85309

C 1.28221 -0.21958 -3.08483

C 0.71426 -2.71267 -2.63472

C -0.61471 -3.48356 -2.62832

C -1.66822 -2.44018 -2.21844

H -2.62744 -2.62486 -2.73285

C -1.94695 -2.37165 -0.71434

C -0.78498 -2.62866 0.22022

C -2.92077 -1.3366 -0.21471

C -3.30238 -2.79274 -0.21858

H -2.64483 -0.93582 0.7672

C -3.64125 -0.31457 -1.07552

H -4.15278 -0.85867 -1.89193

C -4.70503 0.42851 -0.25229

H -4.1537 1.04008 0.48572

C -4.81949 0.14637 2.88553

H -7.32114 -1.06056 2.84212

H -6.86715 -2.46602 1.86047

H -6.13899 -2.22133 3.46136

H -4.52215 -1.62073 1.73675

H -6.08565 -1.22693 -0.05398

H -7.2414 0.84041 1.70157

H -7.86363 0.22665 0.18442

H -6.01342 2.59987 0.66856

H -7.35206 2.49504 -0.48934

H -5.61221 0.36259 -3.00623

H -6.97413 0.05151 -1.9294

H -6.84942 1.60339 -2.77146

H -4.12177 3.05147 -0.70898

H -5.06575 3.27323 -2.18432

H -2.68722 2.64975 -2.68974

H -3.85775 1.52908 -3.35294

H -2.18286 1.78988 0.1159

H -1.00387 2.00436 -1.19454

H -1.02743 0.5009 -0.28356

H -1.26639 0.85449 -3.36784

H -2.6358 -0.2137 -3.67084

H 0.86464 0.68189 -3.5521

H 1.68971 0.11089 -2.10189

H 1.40544 -3.061 -3.41845

H 1.26272 -2.80235 -1.67952

H -0.83665 -3.82137 -3.6503

H -0.60981 -4.37623 -1.98971

H -0.35241 -3.63194 0.0944

H 0.01918 -1.88841 0.08728

H -1.12665 -2.55647 1.26323

H -4.03897 -3.10764 -0.96061

H -3.36993 -3.32947 0.72909

H -5.55131 0.95164 3.04982

H -4.61278 -0.30506 3.8662

H -3.88535 0.60915 2.53697

H 2.14845 -0.56539 -3.66914

C 4.52816 -1.18621 -1.08353

C 5.50228 -1.01661 -2.12319

C 5.57918 0.31539 -2.37665

C 4.63547 0.95888 -1.50888

N 4.00062 0.0265 -0.7339

N 2.57524 2.29978 0.13904

C 3.40286 2.93022 -0.74659

C 3.05191 4.31775 -0.84601

C 1.98015 4.51016 -0.03388

C 1.68858 3.2451 0.57678

C 0.62268 -0.21132 2.86575

N 1.13943 0.6916 1.98044

C 0.4197 1.84231 2.13436

C -0.59887 1.65282 3.12777

C -0.46576 0.38192 3.58953

C 2.28385 -3.54359 2.27981

C 3.13602 -3.80435 1.25515

C 3.38374 -2.5543 0.59409

N 2.68772 -1.55389 1.20928

C 1.99097 -2.13903 2.22898

C 4.38577 2.31807 -1.50399

C 0.66423 3.03734 1.48329

C 4.22755 -2.39912 -0.49168

C 1.04924 -1.51667 3.02736

H 6.05975 -1.82949 -2.57685

H 6.20865 0.83777 -3.0895

H 3.56361 5.03424 -1.4801

H 1.42125 5.42158 0.15167

H -1.3091 2.4157 3.43047

H -1.04606 -0.12915 4.35093

H 1.85929 -4.23115 3.00393

H 3.57469 -4.75112 0.95766

H 4.97063 2.9431 -2.17667

H 0.02207 3.88355 1.72262

H 4.72312 -3.28798 -0.87858

H 0.55318 -2.11953 3.78641

Fe 2.58599 0.36124 0.6266

O 1.27195 -0.01448 -0.43526

S 4.18148 0.78512 2.27649

H 3.79084 2.0569 2.52122

The atomic coordinates of TS-1 module

C 5.27158 -0.2626 3.21315

C 4.31179 -0.5943 2.07654

C 4.85271 -0.04313 0.74155

C 6.07624 -0.84379 0.20628

C 5.6605 -1.37201 -1.17197

C 4.71014 -0.29433 -1.69039

C 5.54637 0.92002 -2.11483

C 3.77821 -0.71854 -2.81665

C 2.7534 0.37666 -3.08992

C 1.95045 0.89099 -1.85309

C 0.89369 -0.15427 -1.5816

C 1.29744 2.20796 -2.32764

C 0.49053 3.0191 -1.37107

C -0.81156 3.32689 -1.45982

C -1.83551 2.78293 -2.39157

C -1.21041 4.31294 -0.39465

C 0.13716 4.79724 0.16056

C 1.12899 3.66381 -0.159

H 2.12401 4.07539 -0.40403

C 1.30411 2.62921 0.95792

C 0.11412 2.38366 1.85792

C 2.12077 1.39844 0.66567

C 2.63896 2.44328 1.62202

H 1.71049 0.48588 1.11612

C 2.87877 1.13283 -0.62057

H 3.48049 2.0316 -0.85014

C 3.85202 -0.04078 -0.43808

H 3.23364 -0.95244 -0.32169

C 4.0146 -2.09087 2.05805

H 6.25951 -0.71926 3.04978

H 5.41778 0.82312 3.3038

H 4.89491 -0.63294 4.17687

H 3.36055 -0.07974 2.28897

H 5.16439 0.99789 0.93654

H 6.37869 -1.65398 0.88161

H 6.95617 -0.19254 0.11704

H 5.11199 -2.32351 -1.06883

H 6.51247 -1.55695 -1.84262

H 4.9458 1.77818 -2.43785

H 6.19692 1.27714 -1.30665

H 6.19402 0.64081 -2.95933

H 3.27391 -1.65908 -2.53188

H 4.34229 -0.9322 -3.73965

H 2.03369 0.04328 -3.8535

H 3.26844 1.2399 -3.53943

H 1.24454 -1.06059 -1.07679

H 0.29983 -0.4074 -2.47155

H -0.04637 0.23579 -0.80704

H 0.69188 1.97487 -3.21899

H 2.1222 2.8528 -2.6879

H -1.45371 1.96362 -3.01563

H -2.6772 2.37209 -1.81068

H -1.81917 5.1358 -0.80231

H -1.83875 3.82161 0.3694

H 0.44551 5.69517 -0.39298

H 0.10821 5.07056 1.2228

H -0.16307 3.28131 2.4294

H -0.75176 2.03115 1.28383

H 0.35612 1.5972 2.58776

H 3.47872 3.04703 1.27137

H 2.65945 2.2308 2.69252

H 4.92514 -2.69578 1.9391

H 3.55704 -2.39436 3.01144

H 3.31857 -2.36863 1.25294

H -2.24887 3.56277 -3.0502

C -3.62738 1.03598 2.05974

C -4.72494 1.96393 2.04984

C -5.25176 1.93195 0.79999

C -4.48177 0.97659 0.05156

N -3.49031 0.45705 0.83105

N -3.00873 -1.08452 -1.52183

C -4.05289 -0.33157 -1.9794

C -4.34922 -0.67536 -3.34104

C -3.47592 -1.65118 -3.69876

C -2.6414 -1.89686 -2.55678

C 0.18595 -2.83496 0.51376

N -0.82679 -2.33478 -0.2578

C -0.76786 -3.01085 -1.44574

C 0.32558 -3.93853 -1.43325

C 0.92285 -3.82575 -0.2174

C 0.06052 -1.17793 3.90368

C -0.89099 -0.29053 4.29499

C -1.76793 -0.1023 3.1742

N -1.3675 -0.88401 2.12606

C -0.25074 -1.54521 2.55187

C -4.7439 0.62745 -1.26061

C -1.6073 -2.81519 -2.52724

C -2.83444 0.77922 3.16313

C 0.47824 -2.45788 1.81102

H -5.03342 2.55576 2.90545

H -6.09311 2.48812 0.39989

H -5.13632 -0.2156 -3.92961

H -3.38435 -2.17023 -4.6473

H 0.58574 -4.58859 -2.26222

H 1.77674 -4.36833 0.17648

H 0.9032 -1.56873 4.46541

H -0.99945 0.21679 5.24814

H -5.56524 1.1364 -1.763

H -1.43286 -3.41367 -3.41986

H -3.04879 1.32608 4.07998

H 1.32676 -2.93719 2.2953

Fe -2.12889 -0.91948 0.26967

O -1.13555 0.4409 -0.24284

S -3.67151 -2.57515 0.94794

H -3.51298 -2.38621 2.27735

The atomic coordinates of TS-2 module

C -6.34388 -1.6927 2.57463

C -5.20631 -0.89811 1.93915

C -5.63701 -0.34083 0.56842

C -6.82877 0.65608 0.67965

C -6.38299 1.94279 -0.02534

C -5.3698 1.46296 -1.06104

C -6.13199 0.83776 -2.23491

C -4.40376 2.52941 -1.5571

C -3.29105 1.89656 -2.38249

C -2.52819 0.72551 -1.7139

C -1.539 1.28742 -0.69125

C -1.745 0.07708 -2.88641

C -0.9153 -1.13331 -2.65387

C 0.42694 -1.27594 -2.84307

C 1.39902 -0.21295 -2.99444

C 0.84902 -2.7025 -2.61737

C -0.48069 -3.47077 -2.60992

C -1.53363 -2.42731 -2.19822

H -2.49414 -2.60809 -2.71003

C -1.8094 -2.35592 -0.69138

C -0.64641 -2.6127 0.24201

C -2.78606 -1.32303 -0.19229

C -3.16635 -2.77852 -0.19888

H -2.50919 -0.92215 0.78885

C -3.50692 -0.30165 -1.05353

H -4.01934 -0.84535 -1.86984

C -4.57035 0.44161 -0.22994

H -4.01869 1.05251 0.50807

C -4.68474 0.15938 2.90762

H -7.18617 -1.0479 2.86435

H -6.73257 -2.45323 1.88238

H -6.00399 -2.20867 3.48306

H -4.38717 -1.60815 1.75978

H -5.95112 -1.21413 -0.03207

H -7.10598 0.85323 1.72348

H -7.72853 0.23955 0.20648

H -5.87829 2.61274 0.69072

H -7.2168 2.50799 -0.4672

H -5.47837 0.37546 -2.98486

H -6.83952 0.06485 -1.90707

H -6.71472 1.61649 -2.74917

H -3.9854 3.06415 -0.68763

H -4.92951 3.286 -2.16266

H -2.55237 2.66216 -2.66867

H -3.72405 1.5413 -3.33123

H -2.04777 1.79209 0.14366

H -0.87847 2.0285 -1.16857

H -0.87973 0.51805 -0.26933

H -1.12884 0.8666 -3.34571

H -2.49827 -0.20222 -3.64871

H 1.0271 0.73588 -3.39715

H 1.65719 0.03914 -1.84322

H 1.54534 -3.05544 -3.39279

H 1.38938 -2.78156 -1.65751

H -0.70448 -3.81246 -3.63003

H -0.47512 -4.35997 -1.9677

H -0.2123 -3.61502 0.11445

H 0.15467 -1.86702 0.11685

H -0.9905 -2.54524 1.28408

H -3.90254 -3.09312 -0.94137

H -3.23117 -3.31821 0.74705

H -5.4171 0.96394 3.07258

H -4.47707 -0.29216 3.88787

H -3.75091 0.62294 2.55951

H 2.35823 -0.50491 -3.43988

C 4.66609 -1.17202 -1.05992

C 5.63933 -1.00368 -2.10206

C 5.7152 0.32823 -2.35635

C 4.7718 0.97116 -1.48617

N 4.14022 0.03984 -0.71011

N 2.70998 2.31402 0.16155

C 3.53721 2.94368 -0.72377

C 3.18644 4.3322 -0.82417

C 2.11497 4.52471 -0.01208

C 1.82422 3.25864 0.59901

C 0.75899 -0.19594 2.88813

N 1.27704 0.70669 2.00535

C 0.55641 1.8548 2.15874

C -0.46537 1.66588 3.15122

C -0.33227 0.39533 3.61235

C 2.41878 -3.53146 2.30237

C 3.27105 -3.79245 1.27787

C 3.51802 -2.54144 0.61676

N 2.81991 -1.54178 1.22946

C 2.12585 -2.12617 2.25002

C 4.51994 2.33086 -1.48147

C 0.8 3.04999 1.50619

C 4.36311 -2.38529 -0.46856

C 1.18455 -1.50228 3.04878

H 6.1981 -1.81627 -2.55495

H 6.34462 0.85078 -3.06931

H 3.69926 5.04906 -1.45712

H 1.55715 5.43662 0.17494

H -1.1757 2.42893 3.45378

H -0.91257 -0.11663 4.37336

H 1.99624 -4.21855 3.0283

H 3.71241 -4.73883 0.98262

H 5.10487 2.95638 -2.15399

H 0.15812 3.89652 1.74617

H 4.86086 -3.27398 -0.85378

H 0.68904 -2.10476 3.80884

Fe 2.71184 0.37265 0.63977

O 1.43375 0.00291 -0.45853

S 4.3192 0.79884 2.30006

H 3.92076 2.06686 2.55162

The atomic coordinates of PC-1 module

C 5.20769 1.08713 -3.28648

C 4.31885 1.13685 -2.04884

C 4.99974 0.4123 -0.86864

C 6.20216 1.21266 -0.28509

C 5.86225 1.46808 1.1882

C 5.03458 0.24453 1.57584

C 5.98346 -0.95109 1.72838

C 4.16942 0.40073 2.81868

C 3.24177 -0.79983 2.97175

C 2.37749 -1.14391 1.71975

C 1.25587 -0.16221 1.66802

C 1.81361 -2.55593 1.99889

C 0.90544 -3.17496 0.99115

C -0.39269 -3.48471 1.13381

C -1.31465 -3.14356 2.25056

C -0.90647 -4.22776 -0.07172

C 0.38202 -4.63308 -0.80413

C 1.42628 -3.58773 -0.36972

H 2.42876 -4.04409 -0.28321

C 1.53542 -2.37273 -1.29812

C 0.29568 -1.9925 -2.07857

C 2.36679 -1.19788 -0.85528

C 2.82592 -2.06106 -2.00293

H 1.92441 -0.23105 -1.12657

C 3.21458 -1.1204 0.40138

H 3.87423 -2.00745 0.42364

C 4.0999 0.13309 0.35866

H 3.41187 0.998 0.44429

C 3.91711 2.57719 -1.74549

H 6.17133 1.58799 -3.10989

H 5.42135 0.0493 -3.57874

H 4.73126 1.58567 -4.14223

H 3.39433 0.58816 -2.28988

H 5.36502 -0.55245 -1.26324

H 6.39021 2.14598 -0.8303

H 7.13124 0.63278 -0.36704

H 5.24307 2.37557 1.29139

H 6.75109 1.60703 1.82088

H 5.46995 -1.88749 1.97381

H 6.56443 -1.13823 0.81654

H 6.6984 -0.75135 2.54054

H 3.57587 1.32883 2.72745

H 4.78932 0.50807 3.72402

H 2.55666 -0.64225 3.81969

H 3.8375 -1.68759 3.24027

H 1.3582 0.78422 1.13871

H 0.50318 -0.19257 2.46087

H -0.5351 -0.48159 0.47838

H 1.3096 -2.51917 2.97919

H 2.68384 -3.22691 2.13208

H -0.7989 -2.69013 3.10758

H -2.05929 -2.41466 1.89126

H -1.52124 -5.0965 0.21237

H -1.55734 -3.57177 -0.67703

H 0.70361 -5.61712 -0.43556

H 0.26899 -4.71747 -1.89212

H -0.01359 -2.79203 -2.76727

H -0.54868 -1.7489 -1.41937

H 0.5006 -1.10271 -2.69232

H 3.68896 -2.70112 -1.80712

H 2.77907 -1.67555 -3.02323

H 4.78808 3.23362 -1.60766

H 3.33783 2.99375 -2.58309

H 3.29704 2.65208 -0.84004

H -1.86076 -4.02982 2.60737

C -3.81386 -0.75979 -2.27573

C -4.94334 -1.62206 -2.48549

C -5.59701 -1.70507 -1.29893

C -4.86476 -0.89514 -0.36535

N -3.7826 -0.33584 -0.97844

N -3.39016 0.75924 1.61681

C -4.5259 0.0449 1.87215

C -4.88132 0.16334 3.2581

C -3.9366 0.94505 3.83951

C -3.00631 1.30712 2.80718

C 0.02195 2.65099 0.09707

N -1.02438 2.03136 0.72274

C -0.98123 2.44019 2.02741

C 0.14112 3.30786 2.23743

C 0.76298 3.44351 1.03654

C 0.01293 1.62755 -3.53416

C -0.90625 0.80532 -4.10531

C -1.84018 0.44934 -3.07618

N -1.49847 1.05753 -1.89891

C -0.36308 1.77473 -2.15779

C -5.22492 -0.72203 0.9584

C -1.88709 2.09198 3.01214

C -2.9148 -0.40126 -3.26313

C 0.34789 2.52936 -1.24131

H -5.18719 -2.09087 -3.43317

H -6.49639 -2.26016 -1.05316

H -5.74618 -0.31057 3.71063

H -3.8544 1.25927 4.87489

H 0.39693 3.75689 3.19149

H 1.6426 4.02835 0.78527

H 0.88142 2.09916 -3.98389

H -0.95937 0.44901 -5.12894

H -6.11658 -1.23843 1.31021

H -1.71694 2.47804 4.01586

H -3.06491 -0.81479 -4.25914

H 1.22136 3.07066 -1.60044

Fe -2.40084 0.83411 -0.12366

O -1.47604 -0.66806 0.27886

S -3.74516 2.6993 -0.59005

H -3.56734 2.67045 -1.92985

The atomic coordinates of PC-2 module

C -6.47879 -1.70575 2.55241

C -5.34119 -0.9112 1.91692

C -5.7719 -0.35394 0.54618

C -6.96364 0.643 0.65741

C -6.51786 1.9297 -0.04761

C -5.50467 1.44985 -1.0833

C -6.2669 0.82465 -2.25716

C -4.53864 2.51634 -1.5794

C -3.42613 1.8835 -2.40476

C -2.66354 0.71281 -1.73628

C -1.67362 1.27388 -0.71346

C -1.88107 0.06453 -2.90941

C -1.05591 -1.14395 -2.67228

C 0.31481 -1.27572 -2.86276

C 1.24083 -0.25071 -3.0665

C 0.7167 -2.71635 -2.63994

C -0.61524 -3.48242 -2.63351

C -1.66806 -2.43935 -2.21961

H -2.62868 -2.61923 -2.73199

C -1.94486 -2.36946 -0.71296

C -0.78073 -2.62541 0.21964

C -2.92087 -1.33608 -0.21463

C -3.30134 -2.7918 -0.22112

H -2.64389 -0.93504 0.76645

C -3.6419 -0.3148 -1.07577

H -4.15437 -0.85848 -1.89211

C -4.70526 0.42849 -0.25221

H -4.15354 1.03939 0.48579

C -4.81964 0.14633 2.88536

H -7.32103 -1.06086 2.84213

H -6.86753 -2.46628 1.8602

H -6.1389 -2.22165 3.4609

H -4.52204 -1.6212 1.73755

H -6.08602 -1.22724 -0.05429

H -7.24081 0.84022 1.70125

H -7.86346 0.22652 0.18428

H -6.01311 2.59966 0.66842

H -7.35166 2.49494 -0.48947

H -5.61331 0.36232 -3.0071

H -6.97446 0.05178 -1.92926

H -6.84962 1.60342 -2.77141

H -4.12017 3.051 -0.70989

H -5.0645 3.27301 -2.18481

H -2.68731 2.64899 -2.69106

H -3.85918 1.52842 -3.35352

H -2.1824 1.77859 0.12159

H -1.01464 2.01661 -1.19065

H -1.01315 0.50461 -0.29281

H -1.2634 0.85259 -3.36948

H -2.63478 -0.21266 -3.6726

H 0.90602 0.75744 -3.31873

H 1.52776 0.04072 -1.45526

H 1.41073 -3.07319 -3.41412

H 1.24935 -2.80248 -1.67838

H -0.8409 -3.82615 -3.65262

H -0.6088 -4.37103 -1.99025

H -0.34646 -3.62782 0.09192

H 0.01895 -1.87847 0.09454

H -1.12525 -2.55898 1.26179

H -4.03794 -3.10593 -0.96343

H -3.36671 -3.33137 0.72489

H -5.55201 0.95091 3.05034

H -4.61191 -0.30515 3.86565

H -3.88584 0.60992 2.53719

H 2.26273 -0.50184 -3.35925

C 4.53137 -1.18582 -1.0825

C 5.5046 -1.01696 -2.124

C 5.58044 0.31526 -2.37838

C 4.63714 0.95838 -1.50886

N 4.00405 0.0268 -0.73297

N 2.57533 2.30079 0.13908

C 3.40255 2.93077 -0.74608

C 3.05154 4.3191 -0.84649

C 1.98001 4.51158 -0.03432

C 1.68901 3.24576 0.57693

C 0.62452 -0.2082 2.86582

N 1.14258 0.69404 1.98455

C 0.42211 1.84104 2.13692

C -0.60055 1.65283 3.12911

C -0.46748 0.38226 3.59025

C 2.28383 -3.5447 2.28018

C 3.1361 -3.80574 1.25569

C 3.38316 -2.5545 0.59483

N 2.68551 -1.55527 1.20751

C 1.99102 -2.13923 2.22792

C 4.38514 2.31786 -1.50395

C 0.66514 3.03681 1.48386

C 4.22832 -2.39858 -0.49094

C 1.04966 -1.51532 3.02636

H 6.06342 -1.82942 -2.57703

H 6.20996 0.83753 -3.09145

H 3.56432 5.03587 -1.47957

H 1.42218 5.42351 0.15249

H -1.31123 2.4158 3.43105

H -1.04818 -0.12995 4.35079

H 1.86112 -4.23177 3.00603

H 3.57739 -4.75213 0.96038

H 4.96999 2.94331 -2.1765

H 0.02326 3.88331 1.72387

H 4.72625 -3.28726 -0.87581

H 0.55411 -2.11772 3.78645

Fe 2.57947 0.36041 0.6211

O 1.28898 -0.01236 -0.49746

S 4.18437 0.7857 2.27718

H 3.78555 2.054 2.5267

# Screening of fermented conditions for *A. oryzae* transformant strain

The strain of the *A. oryzae* transformant harboring *astBC* was inoculated into 10 mL DPY medium (2 % dextrin, 1% polypeptone, 0.5% yeast extract, 0.05% MgSO_4_·7H_2_O, 0.5% KH_2_PO_4_, 0.01% adenine) and was cultured at 28 °C and 200 rpm for 2 days as the seed broth. Then the broth was transferred into 15 Erlenmeyer flasks (500 ml), each containing 100 ml of fermentation medium and growing at 28 °C and 200 rpm. The screening of the fermentation conditions was carried out through bifactor analysis with culture media (rice, ME, PDB, GPY, and maltose media) and fermentation days (3, 5, 7 days for liquid media and 10, 20, 45 days for rice medium, respectively) as variable factors. Rice medium contains 70 g of rice and 105 ml distilled H_2_O on each flask; ME medium contains 2 % malt extract, 1% polypeptone and 2% starch; PDB medium contains 20% potato and 2%dextrose; GPY medium contains 2% starch, 0.5% peptone and 0.2% yeast extract; maltose medium contains 3% starch, 0.15% yeast extract, 0.1% MgSO_4_, 0.25% malt extract, 0.2% KH_2_PO_4_ and 0.4% CaCO_3_ (HPLC analysis see Figure S6-S10).

Figure **S6**| HPLC analysis of metabolites from *A. oryzae* transformant harboring *astBC* fermented in rice medium. (I) metabolites from *A. oryzae* transformant harboring *astBC* cultured for 10 days, (II) metabolites from *A. oryzae* transformant harboring *astBC* cultured for 20 days, (III) metabolites from *A. oryzae* transformant harboring *astBC* cultured for 45 days.

Figure **S7**| HPLC analysis of metabolites from *A. oryzae* transformant harboring *astBC* fermented in ME medium. (I) metabolites from *A. oryzae* transformant harboring *astBC* cultured for 3 days, (II) metabolites from *A. oryzae* transformant harboring *astBC* cultured for 5 days, (III) metabolites from *A. oryzae* transformant harboring *astBC* cultured for 7 days.

Figure **S8**| HPLC analysis of metabolites from *A. oryzae* transformant harboring *astBC* fermented in PDB medium. (I) metabolites from *A. oryzae* transformant harboring *astBC* cultured for 3 days, (II) metabolites from *A. oryzae* transformant harboring *astBC* cultured for 5 days, (III) metabolites from *A. oryzae* transformant harboring *astBC* cultured for 7 days.

Figure **S9**| HPLC analysis of metabolites from *A. oryzae* transformant harboring *astBC* fermented in GPY medium. (I) metabolites from *A. oryzae* transformant harboring *astBC* cultured for 3 days, (II) metabolites from *A. oryzae* transformant harboring *astBC* cultured for 5 days, (III) metabolites from *A. oryzae* transformant harboring *astBC* cultured for 7 days.

Figure **S10**| HPLC analysis of metabolites from *A. oryzae* transformant harboring *astBC* fermented in maltose medium. (I) metabolites from *A. oryzae* transformant harboring *astBC* cultured for 3 days, (II) metabolites from *A. oryzae* transformant harboring *astBC* cultured for 5 days, (III) metabolites from *A. oryzae* transformant harboring *astBC* cultured for 7 days.

Figure **S11**| HPLC-ESI-MS analysis of metabolites from *A. oryzae* transformant harboring *astBC* fermented in ME medium cultured for 3 days. (I) total ion current (TLC) chromatograms; (II) extracted ion chromatogram at *m*/*z* 353 ([M + H – H_2_O]^+^); (III) mass spectrometry of the peak at *t*_R_=27.6 min.

# HPLC-MS analysis of *A. oryzae* transformant strain

Figure **S12**| HPLC- MS analysis. (I) HPLC chromatogram of the extract of the *A. oryzae* transformant harboring *astBC* cultured with ME medium in 3 days, (II) HPLC chromatogram of asperterpenoid E (**2**); (III) extracted ion chromatogram at *m*/*z* 339 ([M + H – H_2_O]^+^) in the extract of the *A. oryzae* transformant harboring *astBC* cultured with ME medium in 3 days; (IV) mass spectrometry of the peak at *t*_R_ = 29.6 min in the extract of the *A. oryzae* transformant harboring *astBC* cultured with ME medium in 3 days; (V) mass spectrometry of asperterpenoid E (**2**).

Figure **S13**| HPLC- MS analysis. (I) HPLC chromatogram of the extract of the *A. oryzae* transformant harboring *astBC* cultured with ME medium in 3 days, (II) HPLC chromatogram of asperterpenoid F (**3**); (III) extracted ion chromatogram at *m*/*z* 355 ([M + H – H_2_O]^+^) in the extract of the *A. oryzae* transformant harboring *astBC* cultured with ME medium in 3 days; (IV) mass spectrometry of the peak at *t*_R_ = 25.2 min in the extract of the *A. oryzae* transformant harboring *astBC* cultured with ME medium in 3 days; (V) mass spectrometry of asperterpenoid F (**3**).

# Structural elucidation of asperterpenoid D (1)

## NMR assignments, UV, IR, ECD, and HRESIMS for asperterpenoid D (1)

Asperterpenoid D (**1**)

**Table S2**| NMR assignments for asperterpenoid D (**1**) (^1^H for 600 MHz and ^13^C for 150 MHz in CDCl_3_)

| No. | *δ*_C_, type | *δ*_H_, (*J* in Hz) *^a^* | ^1^H–^1^H COSY | HMBC | ROESY |
| --- | --- | --- | --- | --- | --- |
| 1 | 47.9, CH_2_ | a: 3.57, d (13.4) | 1b | 2, 3, 6, 10, 11, 12, 15, 19, 21 | 12a, 12b |
|  |  | b: 1.79, d (13.4) | 1a | 6, 10, 11, 15, 19, 21 | 6, 10, 12a, 12b |
| 2 | 162.1, C |  |  |  |  |
| 3 | 126.4, C |  |  |  |  |
| 4 | 33.1, CH_2_ | a: 2.63 | 4b, 5a, 5b | 2, 3, 5, 6 | 20 |
|  |  | b: 2.55, br dd (16.0, 9.8) | 4a, 5a, 5b | 2, 3, 5, 6, 19 |  |
| 5 | 26.0, CH_2_ | a: 1.98, br dd (13.0, 7.5) | 4a, 4b, 5b, 6 | 2, 3, 4, 6, 7 | 20 |
|  |  | b: 1.90, dq (13.0, 9.4) | 4a, 4b, 5a, 6 | 2, 3, 4, 6, 7 |  |
| 6 | 56.8, CH | 2.30, br d (8.7) | 5a, 5b | 2, 3, 4, 5, 7, 8, 9, 20 | 1b, 8a, 8b, 10 |
| 7 | 21.5, C |  |  |  |  |
| 8 | 25.5, CH_2_ | a: 0.61, dd (8.4, 4.2) | 8b, 9 | 2, 6, 7, 9, 10, 20 | 6, 20, 23 |
|  |  | b: 0.37, br t (4.7) | 8a, 9 | 6, 7, 9, 10, 20 | 6, 10, 16 |
| 9 | 29.8, CH | 0.22 | 8a, 8b, 10 | 6, 7, 8, 10, 11, 15, 20 | 15, 20, 21, 23 |
| 10 | 47.7, CH | 1.21 | 9, 15 | 1, 7, 8, 9, 11, 12, 14, 15, 16, 21 | 1b, 6, 8b, 16, 22 |
| 11 | 40.4, C |  |  |  |  |
| 12 | 39.3, CH_2_ | a: 1.61 | 12b, 13a, 13b | 1, 10, 11, 13, 14, 21 | 1a, 1b |
|  |  | b: 1.39 | 12a, 13a, 13b | 1, 11, 13, 14, 21 | 1a, 1b |
| 13 | 35.9, CH_2_ | a: 1.40 | 12a, 12b, 13b | 11, 12, 14, 15, 22 | 21 |
|  |  | b: 1.31 | 12a, 12b, 13a | 11, 12, 14, 15, 18, 22 | 21 |
| 14 | 43.0, C |  |  |  |  |
| 15 | 50.9, CH | 1.21 | 10, 16 | 9, 10, 11, 13, 14, 16, 17, 18, 22, 23 | 9, 18b, 21, 25 |
| 16 | 45.4, CH | 1.77 | 15, 17a, 17b, 23 | 10, 14, 15, 17, 18, 23, 24, 25 | 8b, 10, 22, 24 |
| 17 | 22.4, CH_2_ | a: 1.60 | 16, 17b, 18a, 18b | 14, 15, 16, 18, 23 | 22 |
|  |  | b: 1.45 | 16, 17a, 18a, 18b | 14, 15, 16, 18, 23 | 22, 24, 25 |
| 18 | 40.1, CH_2_ | a: 1.37 | 17a, 17b, 18b | 13, 14, 15, 16, 17, 22 |  |
|  |  | b: 1.00 | 17a, 17b, 18a | 13, 14, 17, 22 | 15 |
| 19 | 171.6, C |  |  |  |  |
| 20 | 20.8, CH_3_ | 0.93, s |  | 6, 7, 8, 9 | 4a, 5a, 8a, 9, 21 |
| 21 | 20.1, CH_3_ | 0.94, s |  | 1, 10, 11, 12 | 9, 13a, 13b, 15, 20 |
| 22 | 17.8, CH_3_ | 0.74, s |  | 13, 14, 15, 18 | 10, 16, 17a, 17b |
| 23 | 28.5, CH | 2.30 | 16, 24, 25 | 15, 16, 17, 24, 25 | 8a, 9 |
| 24 | 23.3, CH_3_ | 0.86, d (6.6) | 23 | 16, 23, 25 | 16, 17b |
| 25 | 15.3, CH_3_ | 0.78, d (6.6) | 23 | 16, 23, 24 | 15, 17b |

*^a^* The indiscernible signals from overlap or the complex multiplicity are reported without designating multiplicity.

Figure S14| UV spectrum of asperterpenoid D (**1**), solvent in CH_3_OH


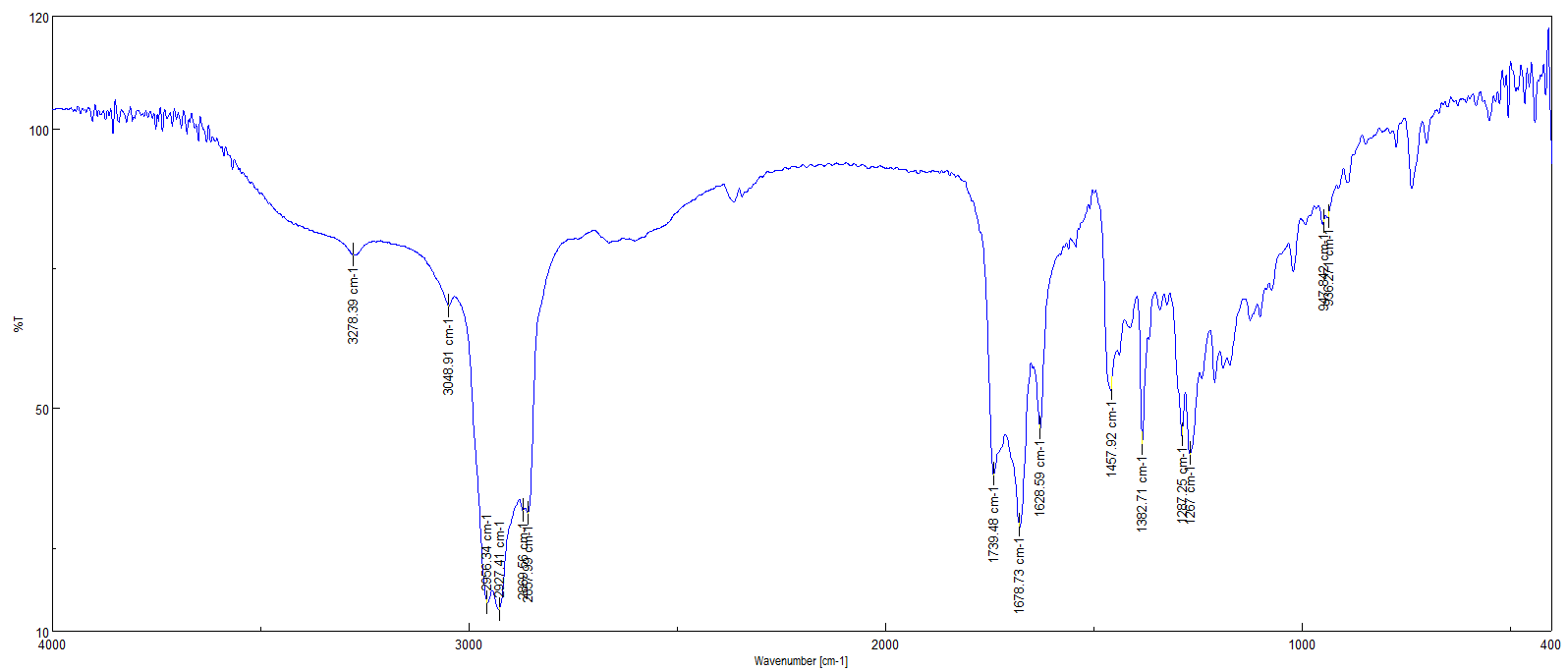


Figure S15| IR spectrum of asperterpenoid D (**1**)


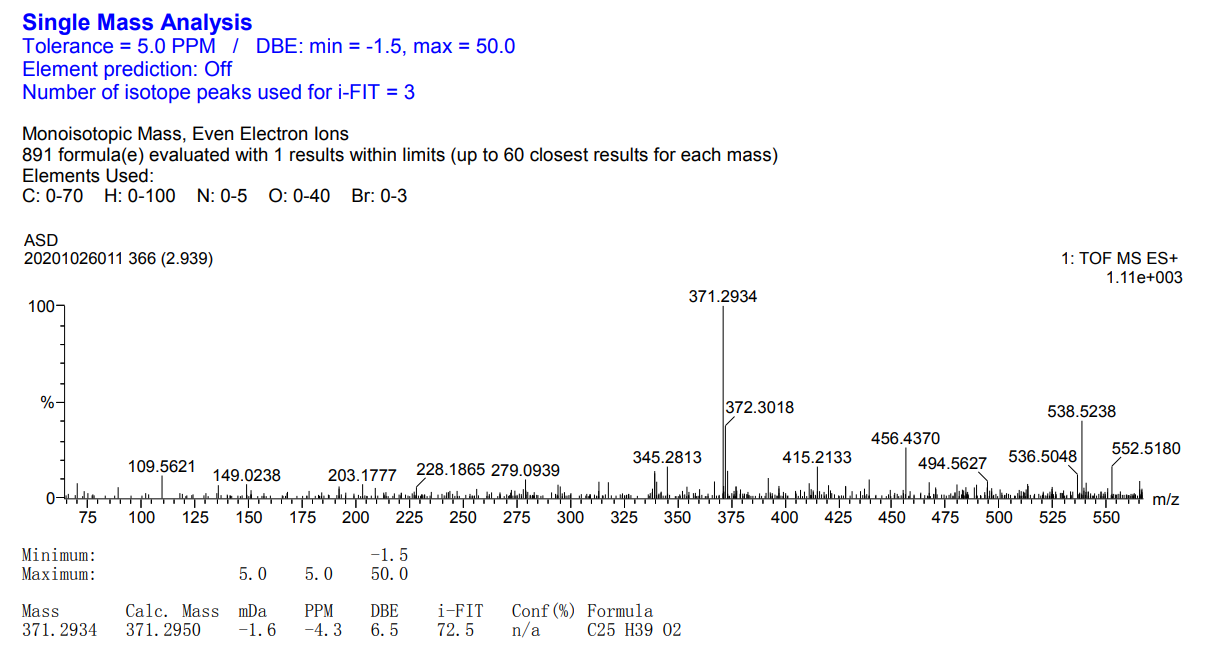


Figure S16| HRESIMS spectrum of asperterpenoid D (**1**)

Figure S17| ECD spectrum of asperterpenoid D (**1**)


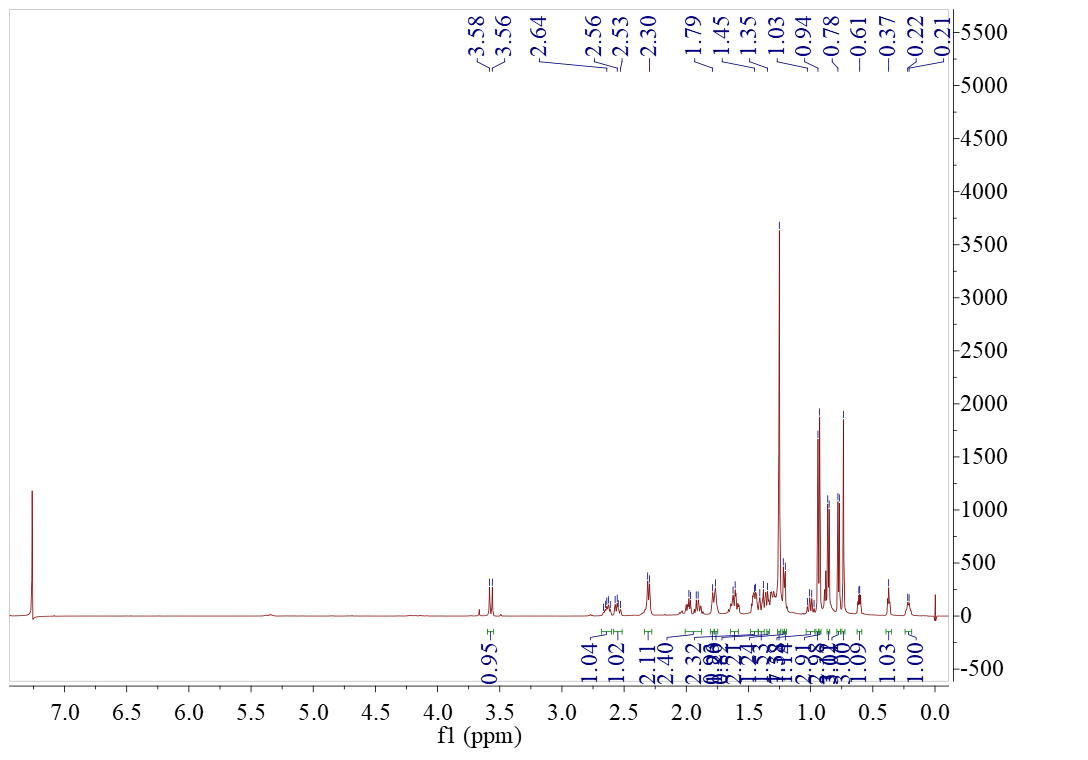


Figure S18| ^1^H NMR spectrum of asperterpenoid D (**1**) in CDCl_3_ at 600 MHz

Figure S19| ^13^C NMR spectrum of asperterpenoid D (**1**) in CDCl_3_ at 150 MHz


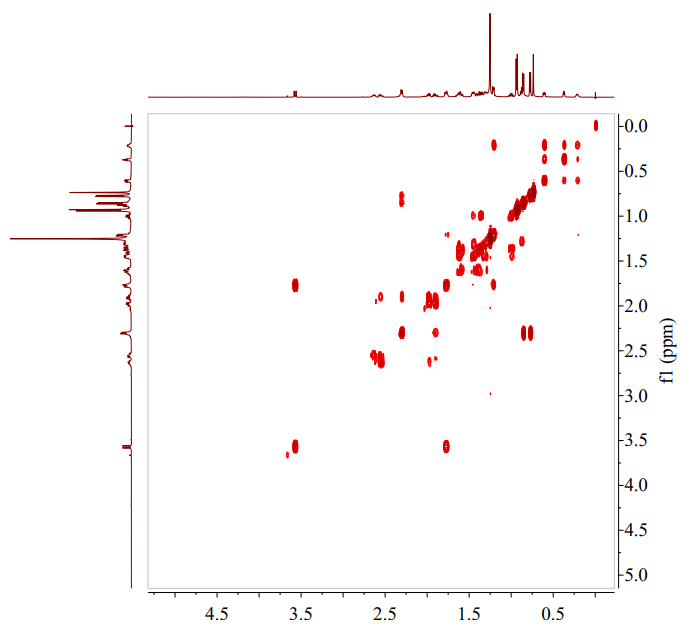


Figure S20| ^1^H-^1^H COSY spectrum of asperterpenoid D (**1**) in CDCl_3_

Figure S21| HSQC spectrum of asperterpenoid D (**1**) in CDCl_3_


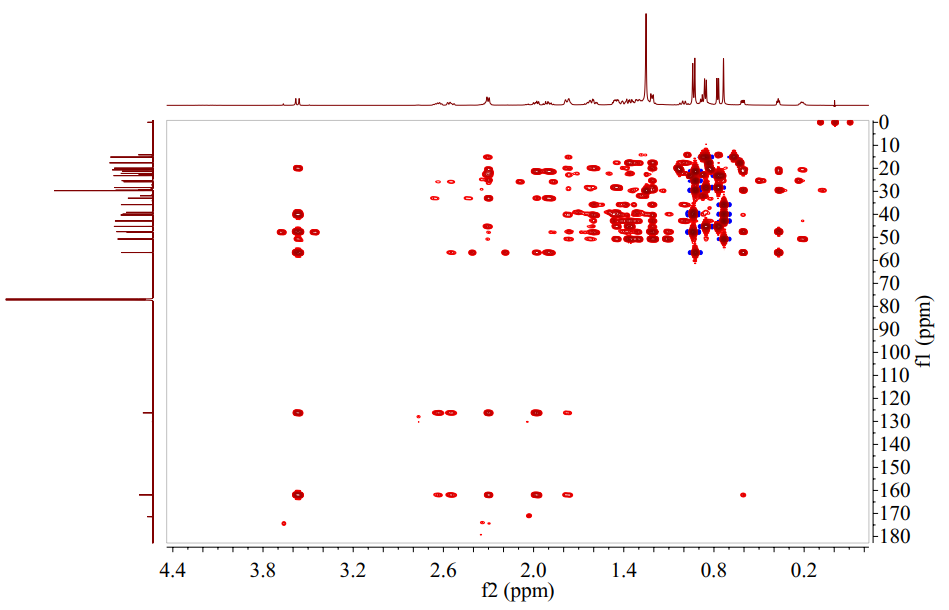


Figure S22| HMBC spectrum of asperterpenoid D (**1**) in CDCl_3_

Figure S23| ROESY spectrum of asperterpenoid D (**1**) in CDCl_3_

## Conformational analysis and ECD-calculation of asperterpenoid D (1)

The molecules of (6*S*, 7*R*, 9*R*, 10*S*, 11*R*, 14*S*, 15*S*, 16*R*)- **1** and (6*R*, 7*S*, 9*S*, 10*R*, 11*S*, 14*R*, 15*R*, 16*S*)-**1** were converted into SMILES codes before their initial 3D structures were generated with CORINA version 3.4. Conformer databases were generated in CONFLEX version 7.0 by using the MMFF94s force-field with an energy window for acceptable conformers (ewindow) of 5 kcal mol^-1^ above the ground state, a maximum number of conformations per molecule (maxconfs) of 100, and an RMSD cutoff (rmsd) of 0.5Å. Then each acceptable conformer was optimized with HF/6-31G(d) method in Gaussian09. Further optimization at the B3LYP/6-31G(d) level determined the dihedral angles. From this, two stable conformers were determined (Table S3 and Figure S24). The optimized conformers were used for ECD calculations, which were performed at the B3LYP/TZVP level. The solvent effects were taken into account by the polarizable-conductor calculation model (PCM, MeOH as the solvent). According to the comparison of experimental ECD of compound **1** and calculated ECD curves, the absolute configuration of compound **1** is determined as 6*S*, 7*R*, 9*R*, 10*S*, 11*R*, 14*S*, 15*S*, 16*R*.

**Table S3**| Stable conformers of (6*S*, 7*R*, 9*R*, 10*S*, 11*R*, 14*S*, 15*S*, 16*R*)-**1** at the B3LYP/6-31G(d) level in MeOH

| Conformer | Contribution % |
| --- | --- |
| C-1 | 66.65 |
| C-2 | 33.35 |


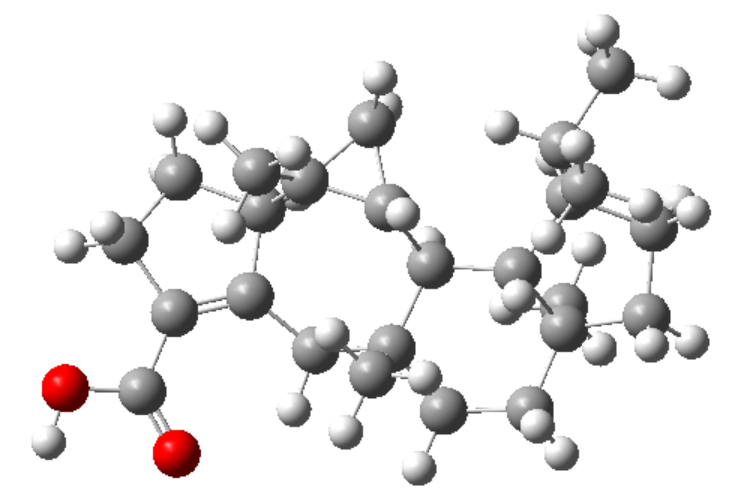

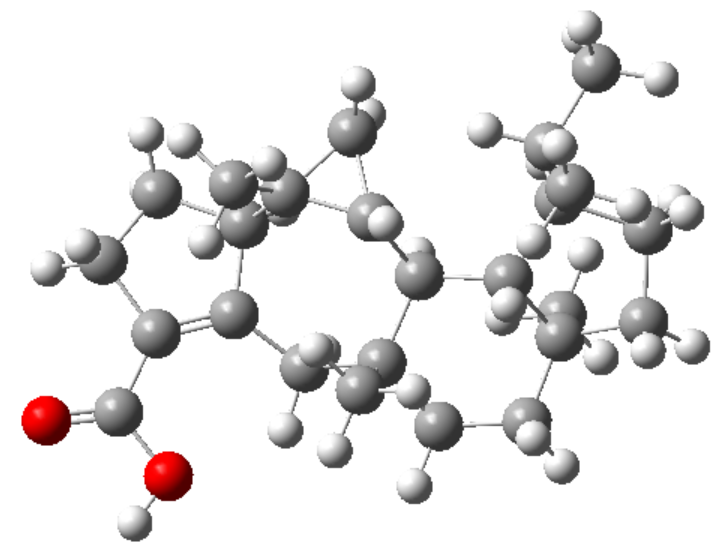


conformer 1 conformer 2

Figure S24| Stable conformers of asperterpenoid D (**1**)

Figure S25| Experimental ECD spectra of asperterpenoid D (**1**) and calculated ECD spectra (UV correction = 0 nm, bandwidth σ = 0.3 eV).

# Structural elucidation of asperterpenoid E (2)

Asperterpenoid E (**2**)

**Table S4**| NMR assignments for asperterpenoid E (**2**). (^1^H for 600 MHz and ^13^C for 150 MHz in CDCl_3_)

| No. | *δ*_C_, type | *δ*_H_, (*J* in Hz) *^a^* | ^1^H–^1^H COSY | HMBC | ROESY |
| --- | --- | --- | --- | --- | --- |
| 1 | 47.0, CH_2_ | a: 2.44, d (13.9) | 1b | 2, 3, 6, 10, 11, 12, 21 | 12a, 19a, 19b |
|  |  | b: 1.71, d (13.9) | 1a | 2, 3, 6, 11, 12, 21 | 6, 10, 12b |
| 2 | 134.7, C |  |  |  |  |
| 3 | 140.6, C |  |  |  |  |
| 4 | 33.7, CH_2_ | a: 2.48 | 4b, 5a, 5b | 2 | 20 |
|  |  | b: 2.33 | 4a, 5a, 5b | 1, 2, 3, 5, 6, 7 | 20 |
| 5 | 26.2, CH_2_ | a: 1.97, br dd (13.1, 7.4) | 4a, 4b, 5b, 6 | 2, 3, 4, 6, 7 | 20 |
|  |  | b: 1.85, dq (13.0, 9.4) | 4a, 4b, 5a, 6 | 4, 6, 7 |  |
| 6 | 54.6, CH | 2.17, br d (9.0) | 5a, 5b | 1, 2, 3, 4, 5, 7, 8, 20 | 1b, 8b, 10 |
| 7 | 22.1, C |  |  |  |  |
| 8 | 25.1, CH_2_ | a: 0.55, dd (8.3, 4.2) | 8b, 9 | 6, 7, 9, 10, 20 | 20, 23 |
|  |  | b: 0.33, dd (5.2, 4.2) | 8a, 9 | 6, 7, 9, 10, 20 | 6, 10, 16, 20 |
| 9 | 29.4, CH | 0.10 | 8a, 8b,10 | 6, 7, 8, 15, 20 | 15, 20, 21, 23 |
| 10 | 47.2, CH | 1.19 | 9, 15 | 1, 8, 9, 11, 12, 14, 15, 16, 21 | 1b, 6, 8b, 16, 22 |
| 11 | 39.2, C |  |  |  |  |
| 12 | 39.3, CH_2_ | a: 1.60 | 12b, 13a, 13b | 1, 10, 11, 13, 14, 21 | 1a |
|  |  | b: 1.33 | 12a, 13a, 13b | 1, 10, 11, 14, 21 | 1b |
| 13 | 35.9, CH_2_ | a: 1.45 | 12a, 12b, 13b | 11, 12, 14, 15, 18, 22 | 21 |
|  |  | b: 1.31 | 12a 12b, 13a | 11, 12, 14, 15, 18, 22 | 21 |
| 14 | 42.9, C |  |  |  |  |
| 15 | 51.0, CH | 1.19 | 10, 16 | 8, 9, 10, 11, 13, 14, 16, 22 | 9, 18b, 21, 25 |
| 16 | 45.3, CH | 1.76 | 15, 17a ,17b, 23 | 10, 15, 17, 23, 24, 25 | 10, 22, 24 |
| 17 | 22.2, CH_2_ | a: 1.61 | 16, 17b, 18a, 18b | 14, 15, 16, 18, 23 | 22 |
|  |  | b: 1.45 | 16, 17a, 18a, 18b | 14, 15, 16, 18, 23 | 25 |
| 18 | 40.0, CH_2_ | a: 1.35 | 17a, 17b, 18b | 13, 14, 15, 16, 17, 22 |  |
|  |  | b: 0.99 | 17a, 17b, 18a | 13, 14, 16, 17, 22 | 15 |
| 19 | 59.0, CH_2_ | a: 4.25, d (11.1) | 19b | 2, 3, 4, | 1a |
|  |  | b: 4.17, d (11.1) | 19a | 2, 3, 4, | 1a |
| 20 | 20.7, CH_3_ | 0.85, s |  | 6, 7, 8, 9 | 4a, 4b, 5a, 8a, 8b, 9, 21 |
| 21 | 20.4, CH_3_ | 0.90, s |  | 1, 10, 11, 12 | 9, 13a, 13b, 15, 20 |
| 22 | 17.6, CH_3_ | 0.73, s |  | 13, 14, 15, 18 | 10, 16, 17a |
| 23 | 28.4, CH | 2.33 | 16, 24, 25 | 15, 16, 17, 24, 25 | 8a, 9 |
| 24 | 23.2, CH_3_ | 0.86, d (6.6) | 23 | 16, 23, 25 | 16 |
| 25 | 15.1, CH_3_ | 0.76, d (6.6) | 23 | 16, 23, 24 | 15, 17b |

*^a^* The indiscernible signals from overlap or the complex multiplicity are reported without designating multiplicity.

Figure S26| UV spectrum of asperterpenoid E (**2**), solvent in CH_3_OH.


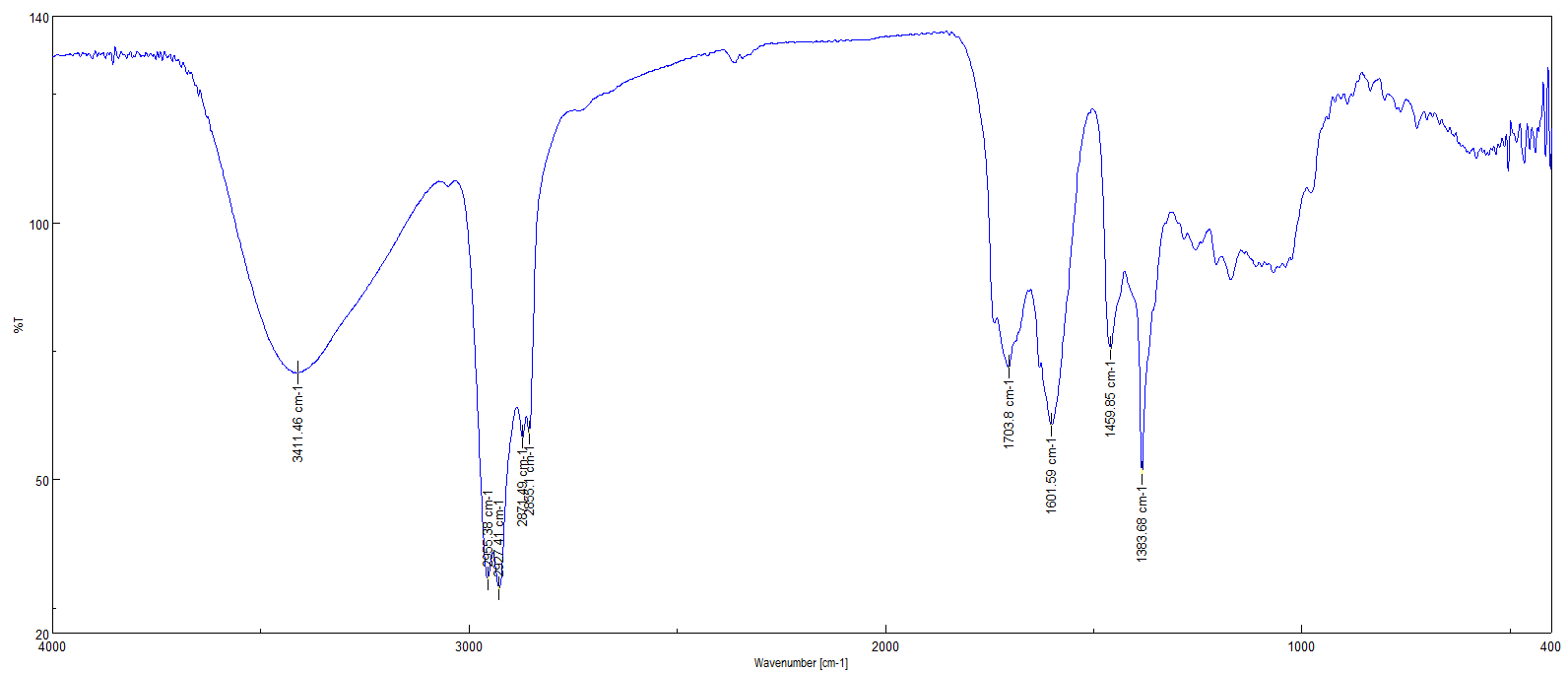


Figure S27| IR spectrum of asperterpenoid E (**2**)

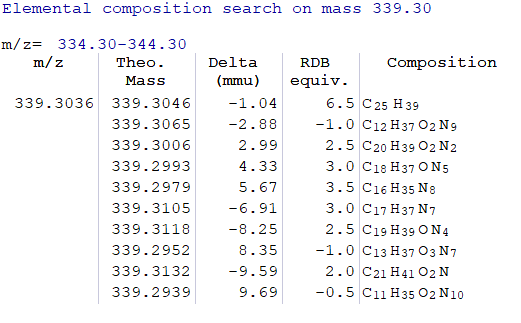


Figure S28| HRESIMS spectrum of asperterpenoid E (**2**).

Figure S29| ECD spectrum of asperterpenoid E (**2**)


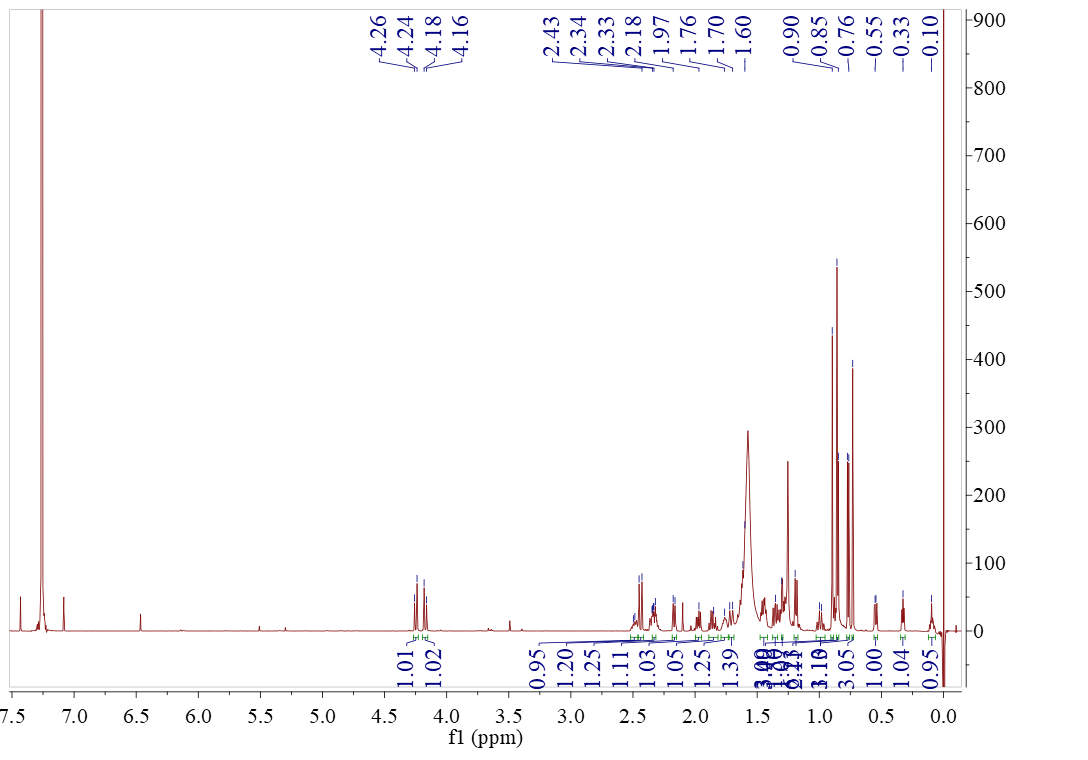


Figure S30| ^1^H NMR spectrum of asperterpenoid E (**2**) in CDCl_3_ at 600 MHz

Figure S31| ^13^C NMR spectrum of asperterpenoid E (**2**) in CDCl_3_ at 150 MHz

Figure S32| ^1^H-^1^H COSY spectrum of asperterpenoid E (**2**) in CDCl_3_


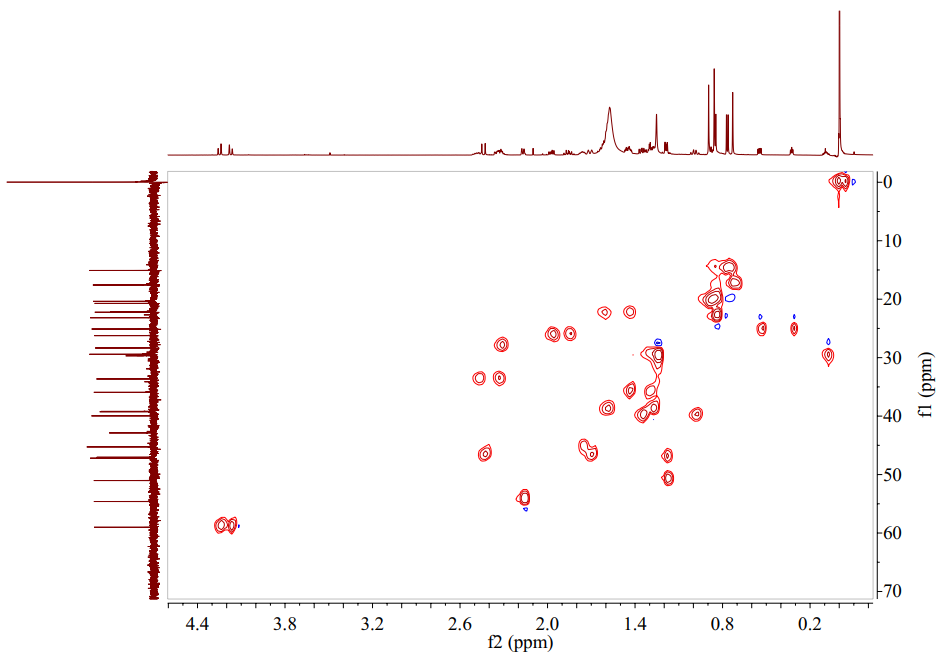


Figure S33| HSQC spectrum of asperterpenoid E (**2**) in CDCl_3_

Figure S34| HMBC spectrum of asperterpenoid E (**2**) in CDCl_3_


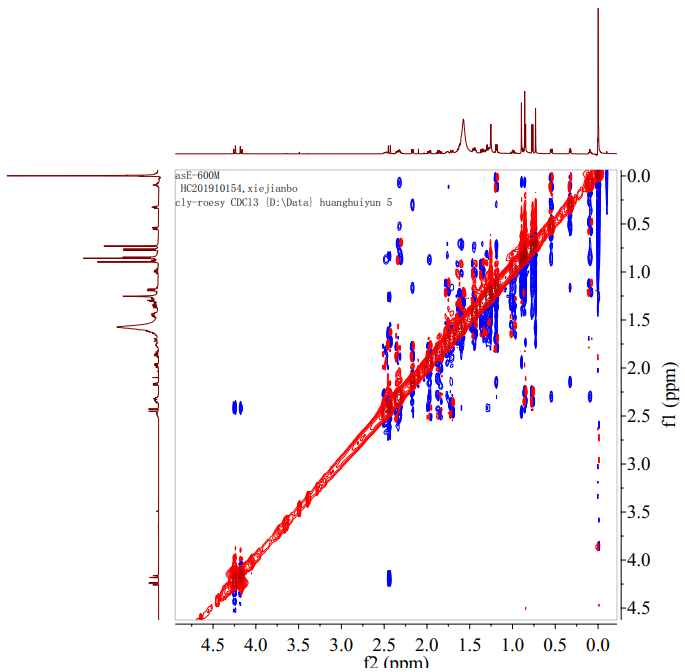


Figure S35| ROESY spectrum of asperterpenoid E (**2**) in CDCl_3_

# Structural elucidation of asperterpenoid F (3)

Asperterpenoid F (**3**)

**Table S5**| NMR assignments for asperterpenoid F (**3**). (^1^H for 600 MHz and ^13^C for 150 MHz in CDCl_3_)

| No. | *δ*_C_, type | *δ*_H_, (*J* in Hz) *^a^* | ^1^H–^1^H COSY | HMBC | ROESY |
| --- | --- | --- | --- | --- | --- |
| 1 | 41.6, CH_2_ | a: 2.94, d (13.7) | 1b | 2, 3, 6, 10, 11, 12, 21 | 12a, 10, 19a |
|  |  | b: 1.45, d (13.7) | 1a | 2, 3, 6, 10, 11, 12, 21 | 6, 12b |
| 2 | 135.6, C |  |  |  |  |
| 3 | 140.7, C |  |  |  |  |
| 4 | 35.5, CH_2_ | a: 2.63 | 4b, 5a, 5b | 2, 3, 5, 6 | 20 |
|  |  | b: 2.13, ddd (15.8, 9.6, 2.6) | 4a, 5a, 5b | 2, 3, 5, 6, 19 |  |
| 5 | 26.6, CH_2_ | a: 1.98, br dd (13.1, 7.2) | 4a, 4b, 5b, 6 | 2, 3, 4, 6, 7 | 20 |
|  |  | b: 1.86, dq (13.1, 9.4) | 4a, 4b, 5a, 6 | 4, 6, 7 |  |
| 6 | 55.4, CH | 2.18, br d (8.8) | 5a, 5b | 1, 2, 3, 4, 5, 7, 8, 9, 20 | 1b, 8b, 10 |
| 7 | 22.6, C |  |  |  |  |
| 8 | 25.8, CH_2_ | a: 0.60, dd (8.4, 4.3) | 8b, 9 | 6, 7, 9, 10, 20 | 20, 23 |
|  |  | b: 0.33, br t (4.9) | 8a, 9 | 6, 7, 9, 10, 20 | 6, 10 |
| 9 | 29.1, CH | 0.06, ddd (10.5, 8.4, 5.6) | 8a, 8b,10 | 6, 7, 8, 10, 15, 20 | 15, 20, 21a, 21b, 23 |
| 10 | 47.7, CH | 1.34, t (11.1) | 9, 15 | 1, 7, 8, 9, 11, 12, 14, 15, 16, 21 | 1a, 6, 8b, 16, 22 |
| 11 | 43.9, C |  |  |  |  |
| 12 | 30.0, CH_2_ | a: 1.91 | 12b, 13a, 13b | 1, 10, 11, 13, 14, 21 | 1a |
|  |  | b: 1.24 | 12a, 13a, 13b | 13, 14, 22 | 1b |
| 13 | 35.7, CH_2_ | a: 1.47 | 12a, 12b, 13b | 11, 12, 14, 15, 22 |  |
|  |  | b: 1.27 | 12a, 12b, 13a | 12, 14, 15, 18, 22 | 21b |
| 14 | 42.8, C |  |  |  |  |
| 15 | 51.4, CH | 1.16, t (11.0) | 10, 16 | 9, 10, 11, 13, 14, 16, 17, 18, 22 | 9, 18b, 21a, 21b, 25 |
| 16 | 45.6, CH | 1.73, tdd (10.5, 4.3, 3.1) | 15, 17a, 17b, 23 | 10, 15, 17, 18, 23, 24, 25 | 10, 22, 24 |
| 17 | 22.2, CH_2_ | a: 1.60 | 16, 17b, 18a, 18b | 15, 18, 23 | 22 |
|  |  | b: 1.44 | 16, 17a, 18a, 18b | 14, 15, 16, 18, 23 | 24, 25 |
| 18 | 39.9, CH_2_ | a: 1.37 | 17a, 17b, 18b | 13, 14, 15, 16, 17, 22 |  |
|  |  | b: 1.00 | 17a, 17b, 18a | 13, 14, 16, 17, 22 | 15 |
| 19 | 59.2, CH_2_ | a: 4.40, d (12.5) | 19b | 2, 3, 4 | 1a |
|  |  | b: 3.98, d (12.5) | 19a | 2, 3, 4 |  |
| 20 | 21.0, CH_3_ | 0.95, s |  | 6, 7, 8, 9 | 4a ,5a, 8a, 9, 21a |
| 21 | 61.3, CH_2_ | a: 3.69, d (10.9) | 21b | 1, 10, 11, 12 | 9, 15, 20 |
|  |  | b: 3.62, d (10.9) | 21a | 1, 10, 11, 12 | 9, 13b, 15 |
| 22 | 17.7, CH_3_ | 0.76, s |  | 13, 14, 15, 18 | 10, 16, 17a |
| 23 | 28.3, CH | 2.27 | 16, 24, 25 | 15, 16, 17, 24, 25 | 8a, 9 |
| 24 | 23.1, CH_3_ | 0.85, d (6.9) | 23 | 16, 23, 25 | 16, 17b |
| 25 | 15.0, CH_3_ | 0.74, d (6.9) | 23 | 16, 23, 24 | 15, 17b |

*^a^* The indiscernible signals from overlap or the complex multiplicity are reported without designating multiplicity.

Figure S36| UV spectrum of asperterpenoid F (**3**), solvent in CH_3_OH.


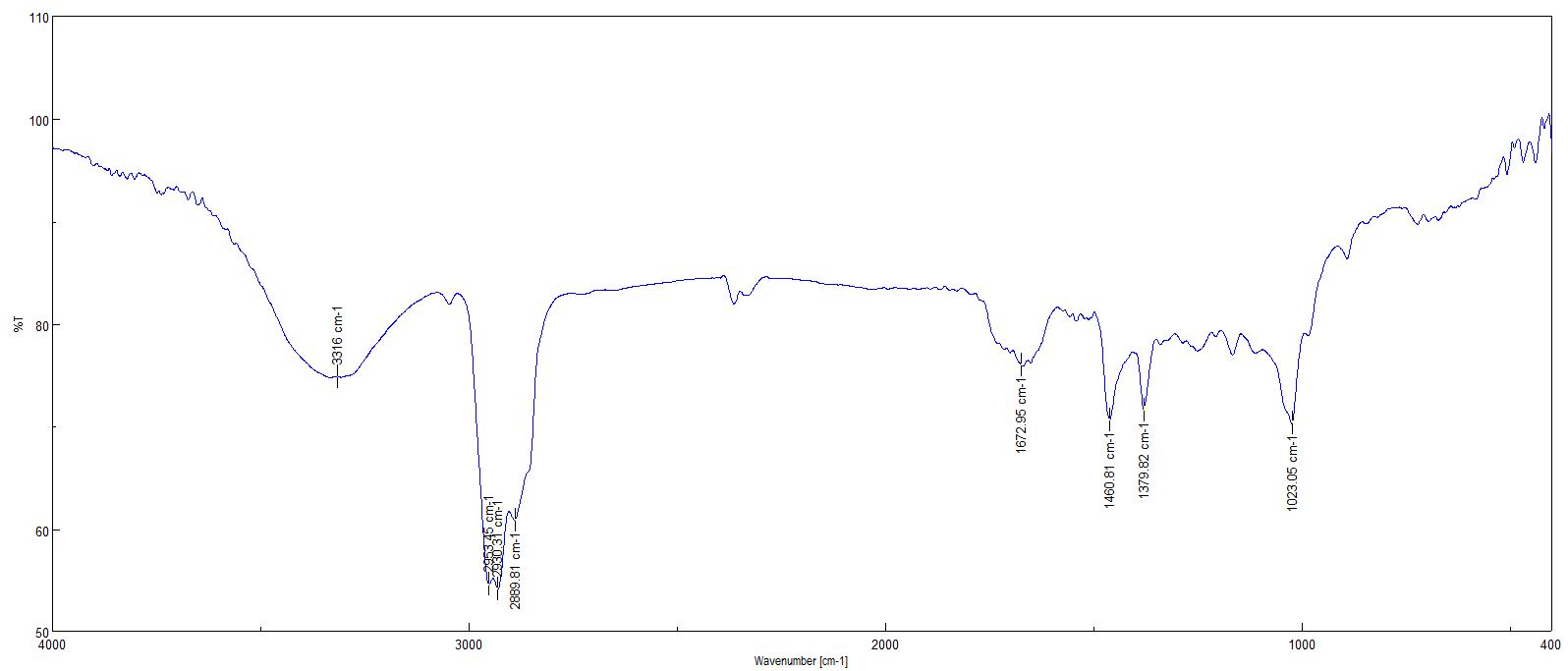


Figure S37| IR spectrum of asperterpenoid F (**3**)


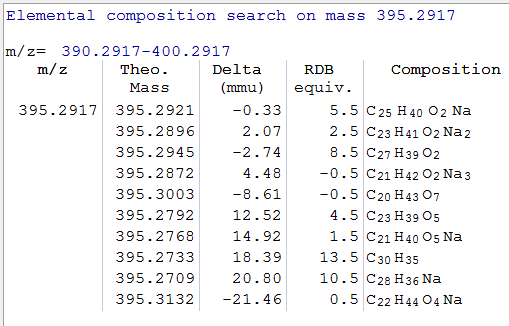


Figure S38| HRESIMS spectrum of asperterpenoid F (**3**)

Figure S39| ECD spectrum of asperterpenoid F (**3**)


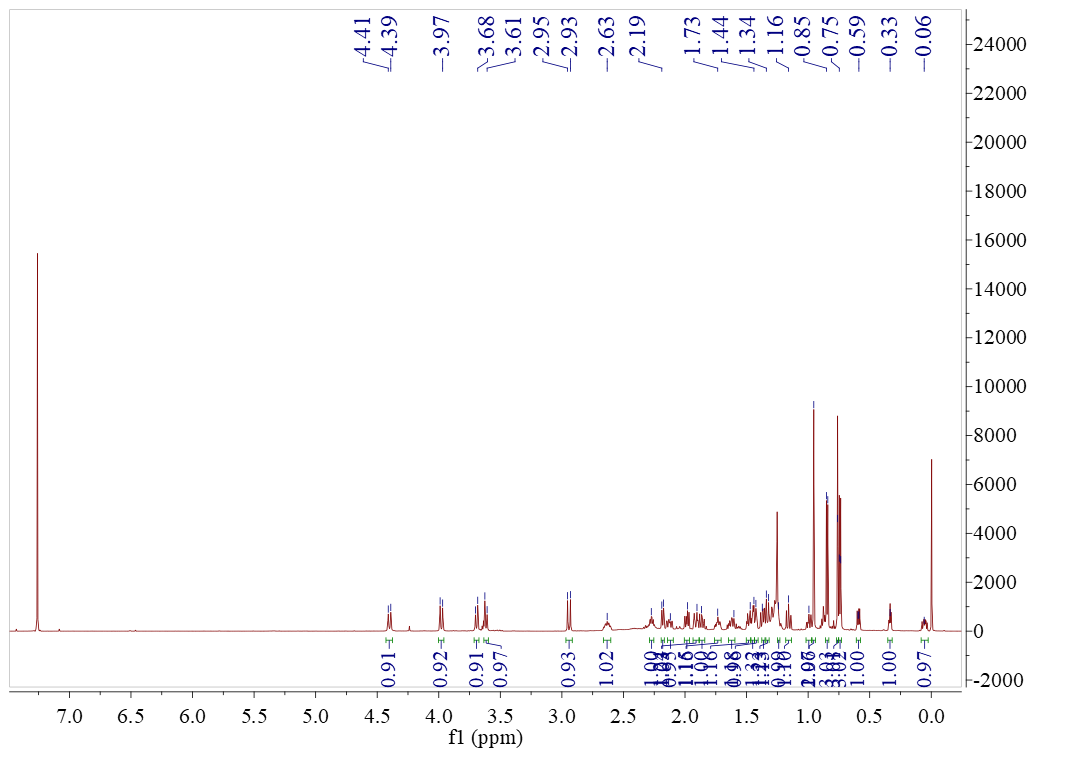


Figure S40| ^1^H NMR spectrum of asperterpenoid F (**3**) in CDCl_3_ at 600 MHz

Figure S41| ^13^C NMR spectrum of asperterpenoid F (**3**) in CDCl_3_ at 150 MHz

Figure S42| ^1^H-^1^H COSY spectrum of asperterpenoid F (**3**) in CDCl_3_

Figure S43| HSQC spectrum of asperterpenoid F (**3**) in CDCl_3_

Figure S44| HMBC spectrum of asperterpenoid F (**3**) in CDCl_3_

Figure S45| ROESY spectrum of asperterpenoid F (**3**) in CDCl_3_

# Heterologous expression, purification, and inhibition assay for *m*PTPB

The coding sequence of *m*PTPB was amplified from the genomic DNA of *M.* *tuberculosis* H37Ra, and then cloned into the expression vector pET28a. Subsequently, the resulting recombinant vector was transformed into *E. coli* BL21(DE3). The transformant was grown in LB medium supplemented with 50 mg L^-1^ kanamycin at 37 ^o^C until OD_600_ reached 0.6-0.8. The gene expression was induced with 0.1 mM isopropyl β-D-thiogalactopyranoside (IPTG) at 18 ^o^C for 16 hours. The cells were harvested by centrifugation at 5000 × *g* for 5 min at 4 ^o^C, and then resuspended in lysis buffer with 0.01% Triton X-100, 5 mL DTT and EDTA-free protease inhibitors cocktail, followed by sonication on ice. After centrifugation at 10000 × *g* for 30 min at 4 ^o^C, the supernatant was collected and subjected to a Ni^2+^-NTA affinity column. After elution with washing buffer (25 mM Tris, 500 mM NaCl, 50 mM imidazole, pH 7.8) to remove the non-specific binding proteins, *m*PTPB was eluted with elution buffer (25 mM Tris, 500 mM NaCl, 350 mM imidazole, pH 7.8). The eluate was concentrated and exchanged with the buffer (25 mM Tris, 100 mM NaCl, pH 7.8) using the Amicon Ultra centrifugal filter. *m*PTPB was analyzed by 10% sodium dodecyl sulfate (SDS)-polyacrylamide gel electrophoresis (PAGE) and its content was determined by Bradford protein assay (Bio-Rad, USA). The purified *m*PTPB was stored at -20 ^o^C.

The phosphatase activity assay of *m*PTPB was performed in triplicate in 96-well microplate in reaction buffer (50 mM Tris, 100 mM NaCl, pH 7.0) using *p*-nitrophenyl phosphate (*p*NPP) as a substrate. The *m*PTPB inhibition was evaluated in a reaction mixture (final volume, 200 μL) containing 1.5 μg *m*PTPB and 50 μM sample, and sodium orthovanadate was tested as the positive control. The reaction mixture was incubated for 10 min at room temperature, followed by addition of *p*NPP to a final concentration of 1.3 mM. The absorbance at 405 nm was measured in the spectrophotometer Infinite 200 PRO (TECAN). The negative control without *m*PTPB was performed to account for the spontaneous hydrolysis of *p*NPP.

IC_50_ with more than 60% of inhibitory activity against *m*PTPB was determined at 0.195-100 μM using two-fold dilution. The data were calculated by fitting the inhibition percentage and inhibitor concentration with Origin 9. Different inhibitor concentrations and different concentrations of *p*NPP were employed to determine the type of inhibition by fitting data to Lineweaver-Burk plot. All assays were performed in triplicate in at least three independent experiments.

**Table S6|** The inhibition rate (%) and IC_50_ value of seven compounds (50 μM) and Na_3_VO_4_ (positive control) against *m*PTPB enzyme.

| Compound | Structure | 50 μM inhibition rate (%) | IC_50_ (μM) |
| --- | --- | --- | --- |
| preasperterpenoid A |  | 31.07 | > 50 |
| asperterpenoid A |  | 98.23 | 2.16 |
| asperterpenoid B |  | 98.91 | 2.50 |
| asperterpenoid C |  | 45.13 | > 50 |
| asperterpenoid D (**1**) |  | 52.81 | 50.34 |
| asperterpenoid E (**2**) |  | 46.79 | > 50 |
| asperterpenoid F (**3**) |  | 47.98 | > 50 |
| Na_3_VO_4_  (positive control) | - | 96.01 | - |
